# The emergence of superficial dermatophytosis due to *Trichophyton indotineae* and *Trichophyton mentagrophytes* genotypes VII and II* in New York: a need for comprehensive testing approaches

**DOI:** 10.1128/jcm.00156-26

**Published:** 2026-04-10

**Authors:** Gabrielle C. Todd, Vinay Vaida, Brittany O'Brien, Sudha Chaturvedi

**Affiliations:** 1 Wadsworth Center Mycology Laboratory, New York State Department of Health 1094, Albany, New York, USA; 2 Department of Biomedical Sciences, College of Integrated Health Sciences, University at Albany 1084, Albany, New York, USA

**Keywords:** *Tm*VII and *Tm*II*, T. indotineae

## Abstract

We report a rapid increase in dermatophyte infections in New York City and surrounding counties from 2022 to 2024. Reported cases include 135 of *Trichophyton indotineae* (*Ti*), 39 of *Trichophyton mentagrophytes* genotype VII (*Tm*VII), and 14 of *T. mentagrophytes* genotype II* (*Tm*II*). These numbers highlight the urgent need for rapid, high-throughput identification, drug-resistance testing, and surveillance of these pathogens. To address these challenges, an automated deep learning model was developed using internal transcribed spacer (ITS) sequences from 28 well-defined genotypes of *Trichophyton interdigitale/T. mentagrophytes* Species Complex (*TiTm*SC). *Ti*, previously classified as *T. mentagrophytes* genotype VIII, is now recognized as a distinct species. The model enabled rapid and accurate identification of all 28 genotypes directly from Sanger sequencing output files. *In vitro* antifungal susceptibility testing (AFST) demonstrated that 57% of *Ti* isolates exhibited high minimum inhibitory concentrations (MICs) to the first-line drug terbinafine, and 38%–52% showed elevated MICs to azoles. In contrast, all *Tm*VII and *Tm*II* isolates had low MICs to both terbinafine and azoles. Whole-genome sequencing (WGS) of a subset of *Ti*, *Tm*VII, and *Tm*II* isolates revealed evidence of both clonal transmission and independent introductions. This integrated approach, combining rapid identification, drug resistance testing, and transmission analysis, is expected to improve patient care and infection control measures.

**IMPORTANCE:**

This report presents a comprehensive laboratory investigation into the emergence and rapid spread of *Trichophyton indotineae* (*Ti*), *Trichophyton mentagrophytes* genotype VII (*Tm*VII), and *T. mentagrophytes* genotype II* (*Tm*II*) in New York City and its surrounding counties. A high-throughput pipeline for the identification of *Trichophyton* species and genotypes is described. Approximately 57% of *Ti* isolates exhibited elevated minimum inhibitory concentrations (MICs) to terbinafine, while 38%–52% demonstrated elevated MICs to azoles, raising significant concern. Whole-genome sequencing revealed evidence of both clonal spread and independent introductions of *Ti*, *Tm*VII, and *Tm*II*. These findings have important implications for patient care and infection control measures.

## INTRODUCTION

Dermatophytosis is a highly prevalent disease that impacts 20%–25% of the population worldwide and, in many parts of the globe, infections are currently being driven by members of the *Trichophyton interdigitale/mentagrophytes* species complex (*TiTm*SC) (1–3). Taxonomic classification of these pathogens has shifted over time; *T. interdigitale* is considered anthropophilic, induces low levels of inflammation, and is the main cause of tinea unguium and tinea pedis, while *T. mentagrophytes* is generally considered zoophilic or geophilic and generates highly inflammatory lesions on other parts of the body (1, 4–6). To date, there are 28 known genotypes of the *TiTm*SC, which cannot be differentiated solely based on phenotypic characteristics and must be distinguished by one to nine single-nucleotide polymorphisms (SNPs) in the internal transcribed spacer (ITS) region (1, 5, 6). Different members of the *TiTm*SC infect different body parts, have different mechanisms of spread, affect populations in different parts of the world, and have different drug resistance profiles (6). Thus, genotyping suspected *TiTm*SC isolates is critical to identify the infection source, monitor spread, and inform clinical treatment options (1).

*T. mentagrophytes* genotype VIII was ultimately given its own species name, *Trichophyton indotineae* (*Ti*), due to its high resistance to the first-line drug terbinafine and its rapid spread throughout India (7). Although this new species was previously categorized as *T. mentagrophytes* and a few cases have been detected in animals (8, 9), it appears to easily spread from person to person, and there is one known instance of likely sexual transmission (10–12). Since its reclassification in 2018, *Ti* has spread globally (8, 13–19). *Ti* causes extensive, pruritic plaques, typically on the trunk, extremities, and groin that are frequently resistant to the first-line drug, terbinafine (15, 18, 20). Infections may require prolonged treatment (e.g., ≥2 months) with second-line drugs such as itraconazole or other antifungals typically reserved for invasive fungal infections (21, 22). Patients of all ages and genders are affected by *Ti* infection. It is hypothesized that the emergence and spread of *Ti* is being driven by inappropriate use and overuse of over-the-counter topical creams containing combinations of antifungals, antibacterials, and high-potency corticosteroids (8, 23–25).

The *Trichophyton mentagrophyte genotype VII* (*Tm*VII) infections, which manifest as pruritic, annular, scaly lesions on areas such as the face, buttocks, and genitals, are mainly diagnosed in men who have sex with men (MSM) (26). These lesions can be particularly severe, causing discomfort and negatively impacting the quality of life for the affected individuals. *Tm*VII infection was first reported in MSM in France and previously in men who traveled to Southeast Asia for sex tourism (26, 27). *Tm*VII is believed to spread through sexual contact and often requires prolonged antifungal treatment. No drug resistance has yet been observed in *Tm*VII to the first-line (terbinafine) or second-line (itraconazole) drugs (28).

While *Trichophyton mentagrophyte* genotype II* (*Tm*II*) is considered a less urgent pathogen than *Ti* or *Tm*VII, it is still a cause for concern due to its ability to cause inflammatory lesions like tinea capitis and tinea corporis (1). Importantly, no resistance against terbinafine or other antifungal drugs has yet been reported in *Tm*II*, providing a strong sense of security about treatment options (1).

We have previously reported the emergence of *Ti* and *Tm*VII in New York City (NYC), United States (US). The first two cases of *Ti* were reported in May 2023, followed by a cohort of 11 cases reported in June 2024 (14, 15). Subsequently, a retrospective analysis identified terbinafine-resistant *Ti* isolates in the US as early as 2017 (18). Similarly, we reported the first case of *Tm*VII in 2024, followed by four cases later that year (28, 29). This emergence is significant as it marks the first documented cases of these pathogens in the US, highlighting the need for increased awareness and understanding of the diseases they cause. To the best of our knowledge, infections with *Tm*II* have thus far not been reported in the US.

Traditional culturing methods are unable to identify *Trichophyton* pathogens at the species and genotype levels, which presents a significant challenge for low-complexity clinical laboratories. Given the large number of genotypes (at least 28) within the *TiTm*SC, sequencing of the ITS region is the ideal method for species and genotype identification (30). Deep learning tools have shown great promise in identifying infectious agents, including dermatophytes (31, 32), underscoring the urgency for adopting new techniques in pathogen identification.

In this study, we have employed comprehensive laboratory approaches, including a deep learning model for rapid and high-throughput *Trichophyton* species and genotype identification directly from Sanger sequencing files, antifungal susceptibility testing (AFST) with a broad range of antifungal drugs to determine resistance profiles, and whole-genome sequencing (WGS) to determine the relatedness and spread of *Ti*, *Tm*VII, and *Tm*II* in the human population. We chose to focus on these three *Trichophyton* species and genotypes because their incidence in the greater NYC area has rapidly increased over the past 3 years. The data provided in this study enhances our understanding of these pathogens and will aid in patient care and infection control measures.

## MATERIALS AND METHODS

### Patient data

This investigation includes 188 unique patients infected with *Ti* or *Tm*VII or *Tm*II*. All patients came from the greater NYC metropolitan area. Patient demographics included age, sex, and county of residence. Only dermatophytes *Ti*, *Tm*VII, and *Tm*II* from the *TiTm*SC were included in this investigation. This study encompassed public health surveillance activities and is exempt according to New York State Department of Health Institutional Review Board guidelines. Informed consent was waived because the data were deidentified.

### Isolate culture and identification

Suspected dermatophyte isolates received from various healthcare facilities in NYC and surrounding counties were cultured on Sabouraud dextrose agar for 3 to 10 days at 30°C, and on potato dextrose agar at 35°C for 7 to 10 days to induce sporulation.

Isolate identification to species and genotype levels was done by DNA sequencing of the ITS region of the ribosomal gene (15). Genomic DNA from each suspected *Trichophyton* isolate was extracted using a QIAamp DNA Mini Kit (Qiagen Inc., Cat #51306). A sterile needle was used to transfer culture to a tube prefilled with beads and lysis buffer. Following a 1-h incubation at 70°C in a thermomixer (Eppendorf, Thermomixer C Model 5382) at 1,000 rpm, tubes were placed in a Precellys homogenizer (Bertin Technologies, Cat #P002511-PEVT0-A.0) at 6,500 rpm 3 × 60 s with a 15 s interval. The lysate was centrifuged briefly to reduce foaming, transferred to a new tube, and DNA was extracted on QIAcube automated DNA extractor (Qiagen Inc., Cat #9001292). Subsequently, the ITS region (ITS1+5.8S+ITS2) was amplified using the primer set V1827 (5′-GGAAGTAAAAGTCGTAACAAGG-3′) and V50 (5′-TCCTCCGCTTATTGATATGC-3′) using AccuTaq LA DNA Polymerase (Sigma, Cat #D5553) and Sanger sequenced by the Wadsworth Center Advanced Genomic Technologies Cluster. For manual analysis of ITS sequences, forward and reverse sequences were trimmed and aligned using Sequencher (Gene Codes Corporation, Ann Arbor, MI, US) and a BLAST search of the consensus sequence was performed at the National Center for Biotechnology Information (https://blast.ncbi.nlm.nih.gov/Blast.cgi) and at the Westerdijk Fungal Biodiversity Institute (https://wi.knaw.nl/page/Pairwise_alignment) to identify the isolate to the species level. Identification down to the genotype level was done by aligning ITS sequences to all 28 known genotypes in the *TiTm*SC (including *Ti*) (5) using CLC Genomics Workbench (version 25.0.3; Qiagen, Inc.). To accurately determine the species and genotype, the entire length of the ITS1-5.8S-ITS2 region (593–596 base pairs depending on the genotype) comprising all unique *TiTm*SC SNPs was used. Importantly, the very first nucleotide of the ITS1 region is essential for determining the species: there is a “G” at this location for all *T. interdigitale* genotypes, and an “A” for all *T. mentagrophytes* genotypes (Fig. S1). Only isolates with a 100% match to one of the 28 known *TiTm*SC ITS sequences were included in further analyses.

### Automated pipeline for *Trichophyton* species and genotype determination

To expedite *Trichophyton* species and genotype identification from ITS sequences, an automated ITS analysis pipeline was developed. Output chromatogram files (.ab1) from Sanger sequencing of the ITS region with forward (V1827) and reverse (V50) primers were directly imported into this pipeline for trimming, aligning, and extraction of the consensus sequence. Subsequently, the consensus sequence was fed into a deep learning Convolution Neural Network (CNN) model trained on 28 curated reference ITS sequences from the *TiTm*SC containing six genotypes of *T. interdigitale* and 21 genotypes of *T. mentagrophytes* (5), and the newly speciated *T. indotineae* (formerly known as *T. mentagrophytes* genotype VIII). The pipeline code can be found in File S1. Also, ITS sequences used to validate the pipeline can be found in GenBank with the accessions PX687495–PX687856 and OR483778–OR483790.

### Antifungal susceptibility testing

AFST was performed by a broth microdilution assay according to Reference Method M38 of the Clinical and Laboratory Standards Institute (33). Minimum inhibitory concentrations (MICs) were determined after 96 h of incubation at 35°C as reported previously (15). The elevated MICs for certain antifungals are based on available epidemiological cutoff values (ECV) (17, 34, 35).

### Whole-genome sequencing and bioinformatic analysis

To determine relatedness within individual *Trichophyton* species and genotypes, 58 *Ti*, 40 *Tm*VII*,* and 14 *Tm*II* isolates whose identification was confirmed by ITS sequencing were sent for whole-genome sequencing at the Wadsworth Center Advanced Genomic Technologies Cluster. Genomic DNA was extracted as described above. Libraries were prepared using Illumina NextSeq 500/550 Mid Output Kit v2.5 (300 Cycles, Illumina, Cat# 20024905) and sequenced on an Illumina NextSeq 500, 550, or 1000 instrument (Illumina). Bioinformatic analyses were performed in CLC Genomics Workbench using the CLC Microbial Genomics Module (version 25.0.3; Qiagen, Inc.) as described previously (15). Briefly, fastq files were imported as Illumina reads and trimmed using a quality score cutoff of 0.5 while removing regions with two or more ambiguous nucleotides. Whole-genome assemblies were prepared *de novo* for each *Ti*, *Tm*VII, and *Tm*II* isolate, and assembly characteristics are summarized in Tables S1 to S3. The *Ti* isolate reads were mapped to TIMM20114 (GCA_023065905.1), *Tm*II* isolate reads to LL-2024a (GCA_047301425.1), and *TmVII* isolate reads to M8436 (GCA_900162065.1) (File S2). Subsequently, identified indels and structural variants were used to guide local read realignment and to detect SNPs with a minimum coverage of 10 and a minimum frequency of 35%. SNPs were used to generate maximum-likelihood phylogenetic trees using a Jukes-Cantor substitution model with 1,000 bootstrap replicates. Sequences of the *SQLE*, *CYP51A,* and *CYP51B* genes were downloaded from NCBI and used as queries to extract gene sequences from each isolate and identify variants. All raw sequencing reads have been uploaded to NCBI’s SRA in PRJNA1046065 (SRR27198731–SRR27198741) and PRJNA1173536 (SRR31013385–SRR31013387 and SRR36030402–SRR36030499).

## RESULTS

### A rise in *Trichophyton* cases in New York

**Fig 1:**
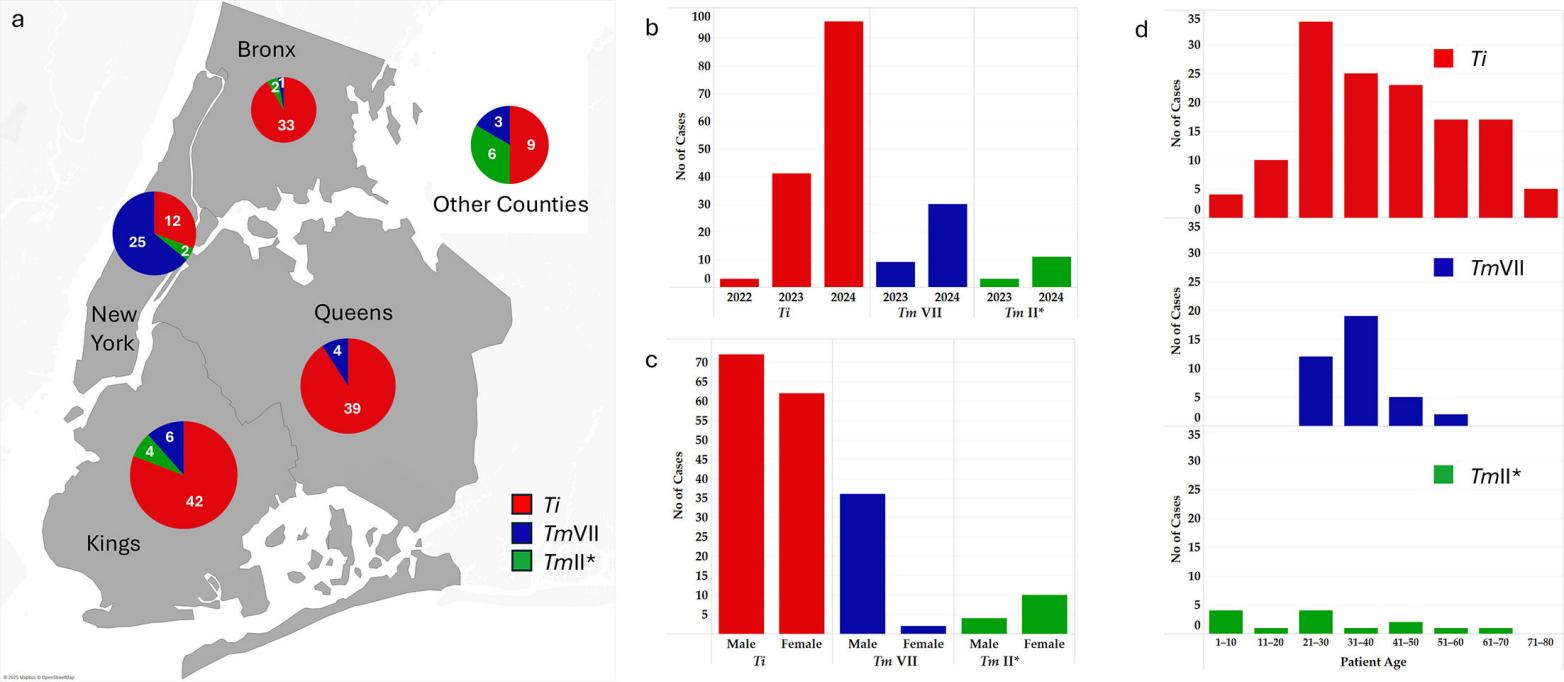
Demographics of *Ti, Tm*VII, and *Tm*II* cases in NYC and surrounding counties. (**a**) A map of NYC counties with overall distribution of *Ti* (red), *Tm*VII (blue), and *Tm*II* (green) cases shown as a pie chart. Most cases are clustered in New York, Bronx, Kings, and Queens counties. Additional cases identified in counties surrounding NYC are grouped in the pie chart outside the NYC map. (**b**) Number of cases identified since 2022. (**c**) Sex distribution of *Ti*, *Tm*VII, and *Tm*II* cases. (**d**) Age range of patients with *Ti*, *Tm*VII, and *Tm*II* infections. Figure 1 is made with Tableau software, https://www.tableau.com.

Over the past 3 years, we have seen a dramatic increase in *Trichophyton* cases in NYC and its surrounding counties (Fig. 1a). We have identified 151 *Ti* isolates representing 135 cases that were concentrated in NYC’s four counties (Kings, Queens, Bronx, and New York), while the other nine cases were from the periphery of NYC. The first *Ti* case was identified in May of 2022, followed by one more case in the same year. The number substantially increased to 36 cases in 2023 and 97 cases in 2024 (Fig. 1b). Almost equal numbers of male and female patients were infected with *Ti* (Fig. 1c), and those infected with *Ti* ranged in age from 4 to 79 (Fig. 1d).

During this period, we also identified 42 isolates of *Tm*VII representing 39 cases. Most of the cases were identified from New York County (25 cases), followed by 14 cases from other NYC counties (Fig. 1a). The first *Tm*VII case was identified in June of 2023, and the total number of cases reached seven by the end of that year. The number of *Tm*VII cases rose substantially in 2024 by 32 cases (Fig. 1b). Male patients outnumbered female patients (Fig. 1c; gender was not specified for one patient) and patients’ age ranged from 25 to 45 (Fig. 1d).

While developing our *TiTm*SC genotyping pipeline, we unexpectedly identified 14 isolates of *Tm*II* representing 14 cases, and these cases were scattered throughout NYC and its surrounding counties (Fig. 1a). Three *Tm*II* cases were identified in 2023, while the other 11 cases were identified in 2024 (Fig. 1b). Interestingly, female patients outnumbered male patients (Fig. 1c), and patients’ ages ranged from 5 to 63 (Fig. 1d).

### ITS sequencing is crucial for *Trichophyton* species and genotype determination

Traditional culturing, microscopy, molecular assays, and MALDI-TOF MS all have limitations in distinguishing among *Trichophyton* species and genotypes (2), highlighting the practical importance of sequencing the entire length of the ITS region. Importantly, the first nucleotide of ITS1 is critical for differentiating *T. interdigitale* (*G*) from *T. mentagrophytes* (A) (Fig. S1). Manual alignment of ITS sequences confirmed a total of 151 *Ti,* 42 *Tm*VII, and 14 *Tm*II* isolates during the study period from May 2022 to December 2024. Additionally, three isolates of *T. interdigitale* I, 159 isolates of *T. interdigitale* II, one isolate of *Tm* III*, two isolates of *Tm*IV, and one isolate of *T. interdigitale* XI were also identified during this time. Nine isolates matched closely to one of the 28 *TiTm*SC genotypes, but they contained a unique SNP and, therefore, could not be assigned as one of the 28 *TiTm*SC genotypes (data not shown).

### An automated ITS analysis pipeline enables rapid and accurate identification of *Trichophyton* species and genotypes

Given the labor-intensive, detailed analyses required to genotype these pathogens via manual alignment of ITS sequences, we developed a pipeline to directly accept Sanger sequencing files as input, trim and align the sequences, and then utilize a deep learning CNN model with 28 curated ITS sequences of the *TiTm*SC (5) to assign a *Trichophyton* genotype (Fig. S2a). The model was evaluated with 382 clinical isolates of *Trichophyton* received in the laboratory from May 2022 to December 2024. The deep learning CNN model accurately predicted *Trichophyton* species and genotypes with 98.7% concordance to the manual alignment approach, confirming its excellent performance (Fig. S2b). Nine isolates were closely matched to one of the 28 genotypes but had a single unique SNP difference from the closest reference sequence. Five isolates had an underlying background in the chromatogram files (Fig. S2c) and thus did not pass through the pipeline, but could be manually inspected and, once a consensus sequence was extracted, were able to be assigned a genotype via the pipeline. In addition, the pipeline allowed simultaneous analysis of multiple ITS sequences, making the analysis much faster and more efficient.

### Emerging antifungal resistance in *TiTm*SC

One hundred thirty-three isolates of *Ti,* 39 isolates of *Tm*VII, and 10 isolates of *Tm*II* were tested against a panel of antifungal drugs by a broth microdilution assay for molds according to Reference Method CLSI M38 of the CLSI guidance document (33) (Table 1). Based on available ECV for certain drugs (17, 34, 35), more than half of the *Ti* isolates had elevated MICs to the first-line drug, terbinafine (57%), followed by voriconazole (52%), fluconazole (38%), and ketoconazole (31%). Elevated MIC to the second-line drug, itraconazole, was minimal (5.3%). Furthermore, *Ti* isolates generally fell into three distinct categories of terbinafine MICs: low MICs (≤0.03 µg/mL; 41%), moderate MICs (0.5–2 μg/mL; 17%) or high MICs (≥16 µg/mL; 40%) (Table 1). The squalene epoxidase-encoding *SQLE* gene was extracted from *de novo* assembled whole genomes of *Ti* and all but one of the isolates with moderate MICs contained the mutation L393S, while all isolates with high MICs contained the mutation F397L (Table 1). The *SQLE* mutations A448T, F415Y, R413G, S443P, and F415C were also observed but did not appear to impact terbinafine resistance (data not shown).

**TABLE 1:** MIC/MEC distribution of *Trichophyton* spp. against 13 antifungal drugs^*a*^

No. of *Ti* isolates with MIC/MEC (µg/mL)																
Drugs	Number isolates tested	≤0.03	0.06	0.125	0.25	0.5	1	2	4	8	16	32	≥64	Range	MIC_50_	MIC_90_
AMB	133				9	81	38	5						0.25–2	0.5	1
AND	133	132	1											≤0.03–0.06	≤0.03	≤0.03
MCF	133	130	3											≤0.03–0.06	≤0.03	≤0.03
CSP	108	108												≤0.03	≤0.03	≤0.03
IBX	81	79	2											≤0.03–0.06	≤0.03	≤0.03
PSC	133	41	38	29	22	2	1							≤0.03–1	0.06	0.25
VRC	133	1	14	49	20	17	27	5						≤0.03–2	0.25	1
FLC	133							3	9	12	58	27	24	2 to ≥64	16	≥64
ITC	133	38	33	25	30	7								≤0.03–0.5	0.06	0.25
KTC	108		15	15	37	23	15	3						0.06–2	0.25	1
ISA	124		1	3	4	11	42	28	15	20				0.06–8	2	8
GRF	133			1		1	28	84	17	1			1	0.125–64	2	4
TRB	133	55		1	1	3	11	9			1	2	50	≤0.03 to ≥64	1	≥64
						SQLE L393S					SQLE F397L					
No. of *Tm*VII isolates with MIC/MEC (µg/mL)																
AMB	39					8	23	6	2					0.5–4	1	2
AND	39	39												≤0.03	≤0.03	≤0.03
MCF	39	39												≤0.03	≤0.03	≤0.03
CSP	32	32												≤0.03	≤0.03	≤0.03
IBX	30	29	1											≤0.03–0.06	≤0.03	≤0.03
PSC	39	25	11	3										≤0.03–0.125	≤0.03	0.06
VRC	39	5	16	16	2									≤0.03–0.25	0.06	0.125
FLC	39							4	3	17	10	2	3	2–64	8	32
ITC	39	21	11	4	3									≤0.03–0.25	≤0.03	0.125
KTC	32	3	1	7	13	4	3	1						≤0.03–2	0.25	1
ISA	39			5	23	11								0.125–0.5	0.25	0.5
GRF	39			1		17	18	3						0.125–2	1	1
TRB	39	39												≤0.03	≤0.03	≤0.03
No. of *Tm*II* isolates with MIC/MEC (µg/mL)																
AMB	10					5	5							0.5–1	0.5	1
AND	10	10												≤0.03	≤0.03	≤0.03
MCF	10	10												≤0.03	≤0.03	≤0.03
CSP	10	10												≤0.03	≤0.03	≤0.03
IBX	10	10												≤0.03	≤0.03	≤0.03
PSC	10	10												≤0.03	≤0.03	≤0.03
VRC	10	7	3											≤0.03–0.06	≤0.03	0.06
FLC	10						1	1	1	6	1			1–16	8	8
ITC	10	10												≤0.03	≤0.03	≤0.03
KTC	10	4	2	2	1	1								≤0.03–0.5	0.06	0.25
ISA	10	3		1	5	1								≤0.03–0.5	0.25	0.25
GRF	10			1	4	4	1							0.125–1	0.25	0.5
TRB	10	10												≤0.03	≤0.03	≤0.03

^*a*^ MIC, minimum inhibitory concentration; MEC, minimum effective concentration; AMB, amphotericin B; AND, anidulafungin; MCF, micafungin; CSP, caspofungin; IBX, ibrexafungerp; PSC, posaconazole; VRC, voriconazole; FLC, fluconazole; ITC, itraconazole; KTC, ketoconazole; ISA, isavuconazole; GRF, griseofulvin; TRB, terbinafine; SQLE, squalene epoxidase. AMB MIC was read as the lowest antifungal concentration, showing 100% inhibition of growth relative to the positive control well. AND, MCF, CSP, IBX MECs were read as the lowest concentration of drug that led to the growth of small, rounded, compact hyphal forms as compared to the hyphal growth seen in the growth control well. All other drugs were read at the lowest antifungal concentration, showing 80% inhibition of growth relative to the positive control well. Light gray shading indicates ECV (when known). Dark gray shading indicates mutations in SQLE correlated with TRB resistance. MIC_50_ and MIC_90_ represent the MICs of antifungal agents required to inhibit the growth of 50% and 90%, respectively, of a *Ti, Tm*VII, and *Tm*II* isolates in a tested population.

To our knowledge, no ECVs have been determined for *Tm*VII or *Tm*II*. Based on *Ti* ECVs, only a small number of *Tm*VII isolates had elevated MICs against voriconazole (5%), fluconazole (13%), or ketoconazole (21%). For *Tm*II*, except for one isolate with elevated MIC for ketoconazole (10%), all other *Tm*II* isolates had low MICs for all other drugs. Importantly, both *Tm*VII and *Tm*II* exhibited low MICs to the first-line drug, terbinafine, as well as to the second-line drug, itraconazole.

### WGS revealed clonal and non-clonal spread of *Trichophyton* species and genotypes

To investigate the spread of *Ti*, *Tm*VII, and *Tm*II* among patients residing in NYC and its periphery, WGS was conducted on select isolates from each species (*Ti* [58], *Tm*VII [40], and *Tm*II* [14]). The average genome size for *Ti*, *Tm*VII, and *Tm*II* isolates was 22.13, 22.58, and 22.63 Mbp, respectively, with coverage depth ranging from 44 to 263× (Tables S1 to S3).

**Fig 2:**
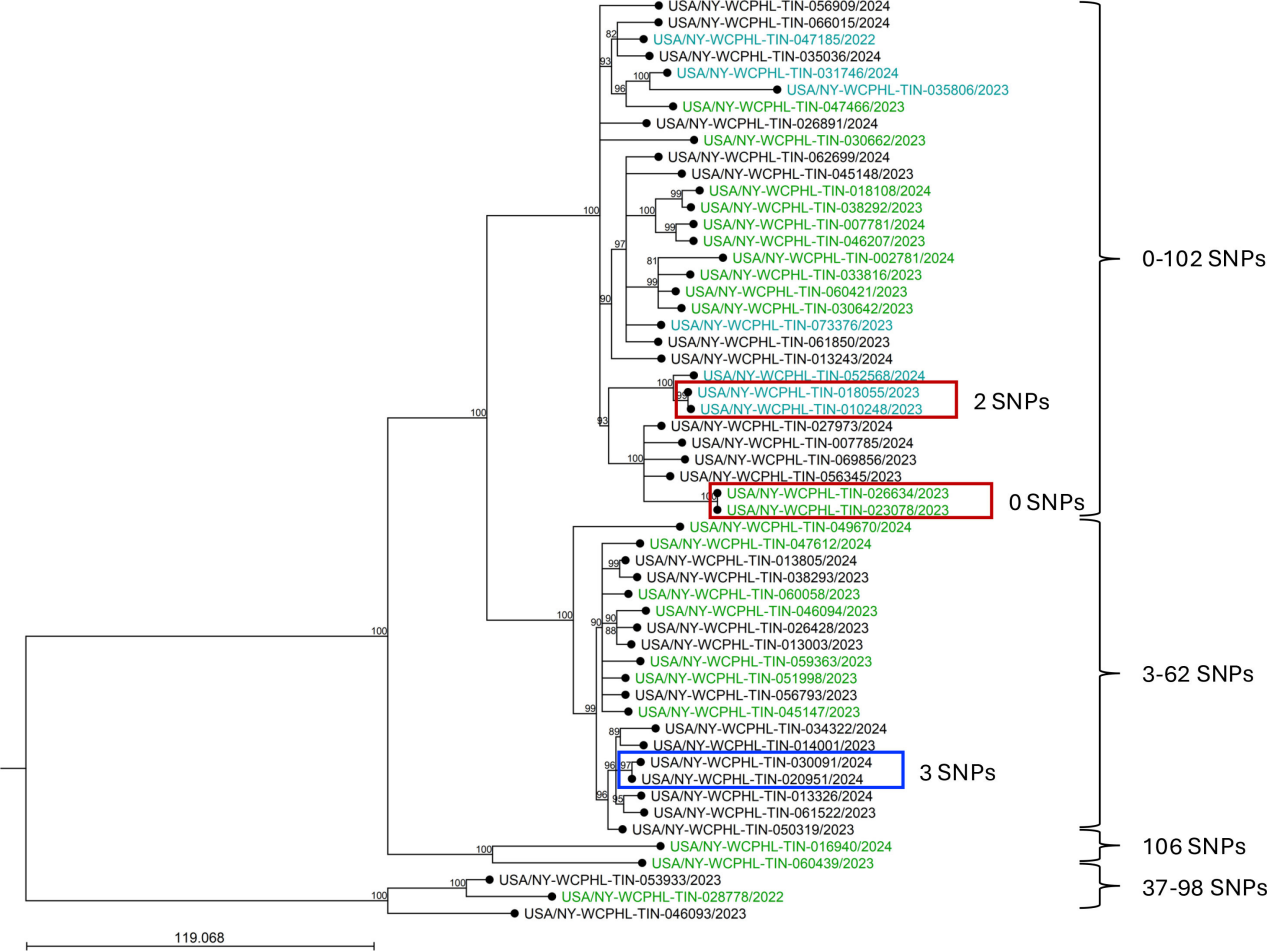
Maximum-likelihood SNP tree of *Ti* isolates. *Ti* reads were mapped to the reference strain TIMM20114, and a phylogenetic tree was constructed using a maximum-likelihood algorithm with a Jukes-Cantor substitution model and 1,000 bootstrap replicates. A bootstrap cutoff of 80% was used, and bootstrap values are indicated at nodes. The scale bar indicates the number of expected substitutions between isolates. All *Ti* isolates formed four distinct clusters. SNP ranges for each cluster are shown next to the brackets on the right. Two confirmed cases of household transmission, identified in a previous investigation (15), are boxed in red, and the third instance of likely local transmission is boxed in blue. Isolates with SQLE mutations F397L are in green and L393S are in teal.

Mapping of 55 *Ti* isolate reads to the reference strain TIMM20114 identified four major clusters, with SNPs ranging from 0 to 415 and a median of 107. SNP differences within each cluster were <106 (Fig. 2). Additionally, several pairs of isolates showed 0–3 SNP differences. Due to the lack of detailed epidemiological data for most sequenced *Ti* isolates, the potential influence of patient geographic proximity on *Ti* spread was assessed. Seventeen *Ti* isolates from 17 patients residing within a single zip code were analyzed. The analysis showed that *Ti* isolates formed into three distinct clusters with two isolates not belonging to any cluster (Fig. S4). SNP differences among these isolates ranged from 3 to 138.

**Fig 3:**
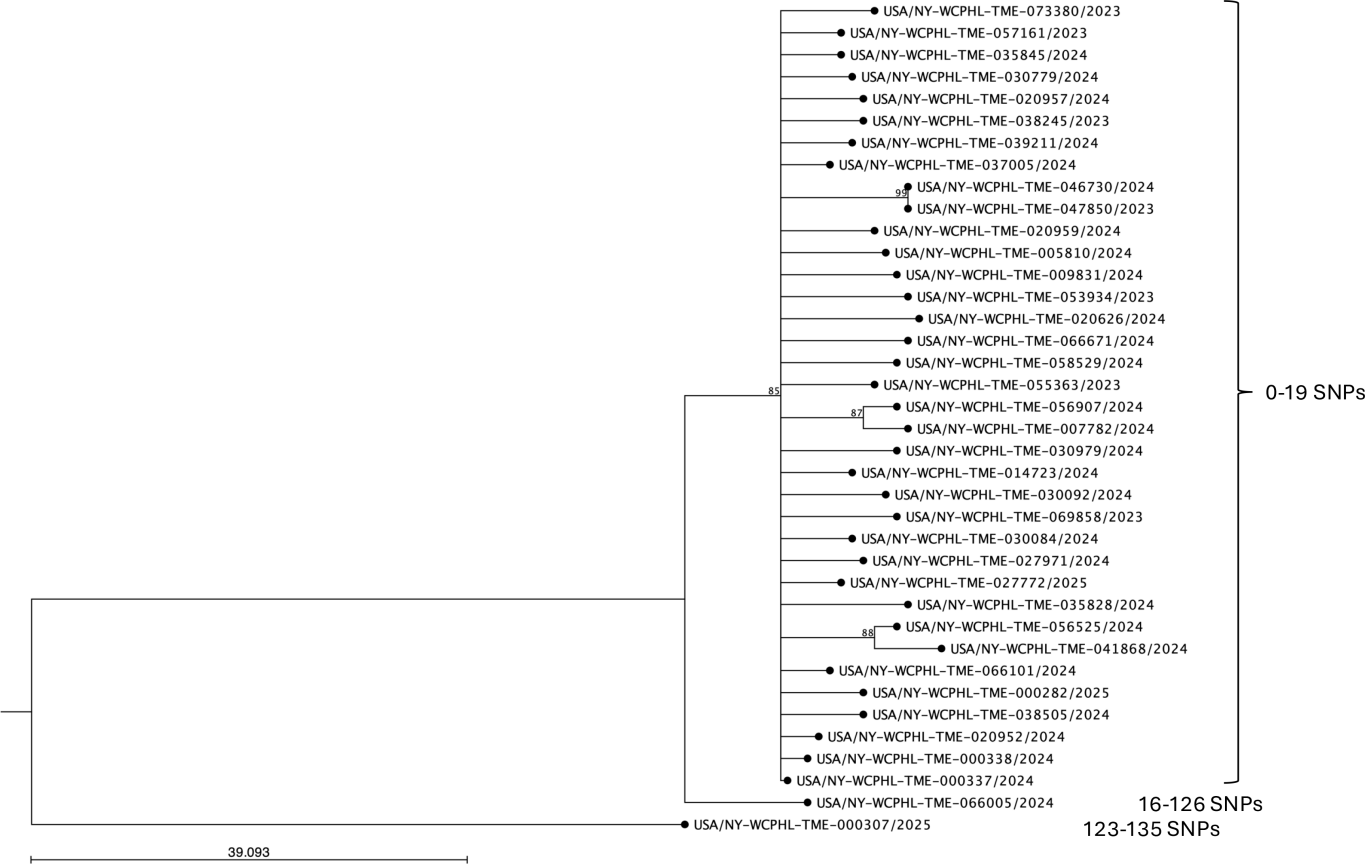
Maximum-likelihood SNP tree of *Tm*VII isolates. *Tm*VII reads were mapped to the reference strain M8436, and a phylogenetic tree was constructed using a maximum-likelihood algorithm with the Jukes-Cantor substitution model and 1,000 bootstrap replicates. A bootstrap cutoff of 80% was applied, and bootstrap values are indicated at the nodes. The scale indicates the number of expected substitutions between isolates. All but two isolates of *Tm*VII grouped into a single cluster with <19 SNP differences among them, suggesting possible local spread in the greater NYC area. SNP ranges in each cluster are provided next to the brackets on the right.

The *Tm*VII isolates analyzed in this study were mapped to the GenBank assembly strain M8436, which was confirmed as *Tm*VII using the genotyping pipeline developed in this study. In contrast to the findings of *Ti*, all but two *Tm*VII isolates clustered in the phylogenetic tree with fewer than 19 SNP differences (Fig. 3). One isolate was positioned outside the main cluster, exhibiting 16–27 SNP differences from the main group. The second isolate originated from a patient in a county with no other reported cases and was highly divergent from other *Tm*VII isolates, with SNP differences ranging from 123 to 135.

**Fig 4:**
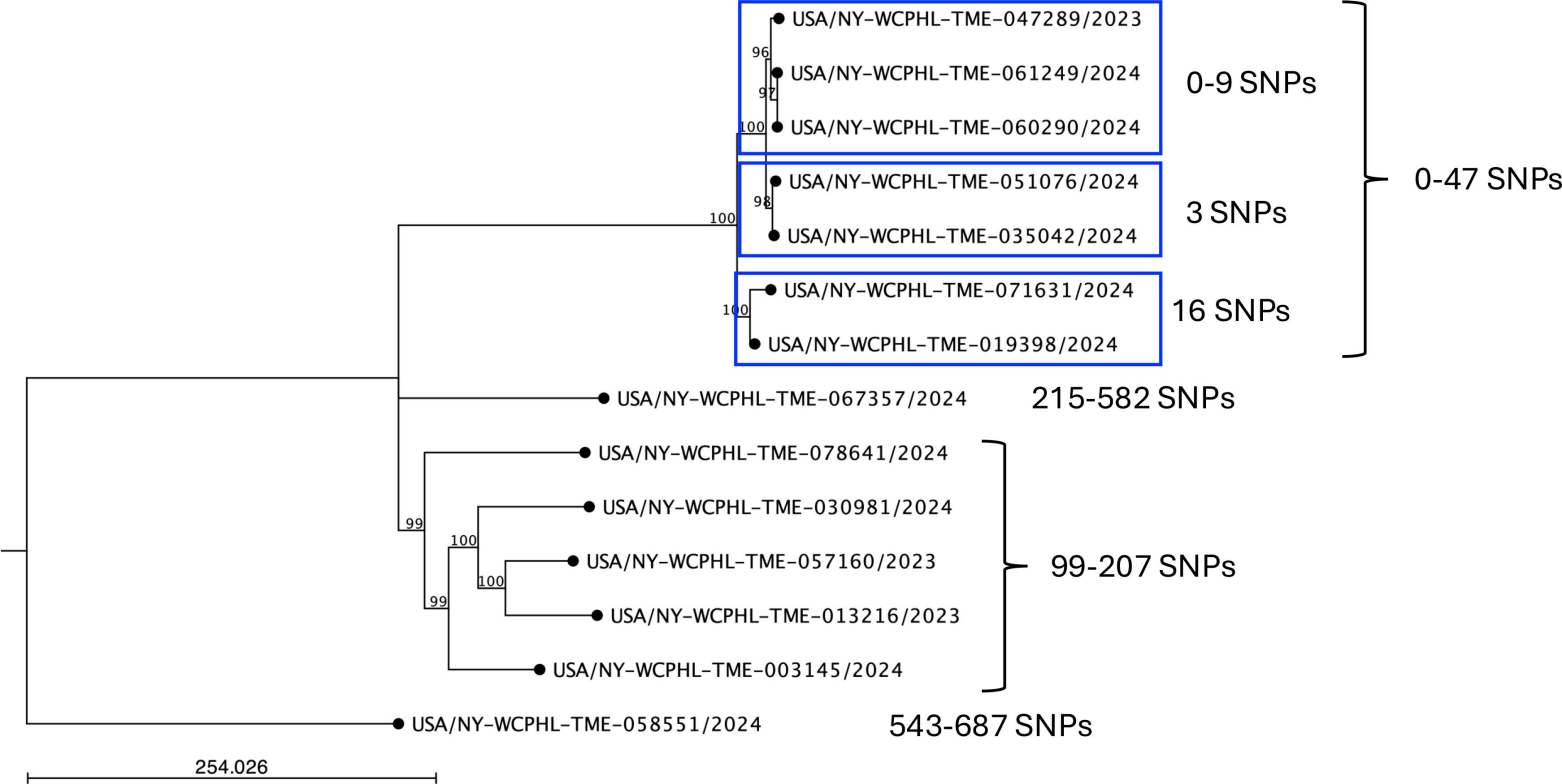
Maximum-likelihood SNP tree of *Tm*II* isolates. *Tm*II* reads were mapped to the reference strain LL-2024a, and a phylogenetic tree was constructed using a maximum-likelihood algorithm with the Jukes-Cantor substitution model and 1,000 bootstrap replicates. A bootstrap cutoff of 80% was applied, with bootstrap values shown at each node. The scale bar represents the number of expected substitutions between samples. The *Tm*II* isolates formed four distinct clusters. SNP ranges in each cluster are provided next to the brackets on the right. Blue boxes indicate potential cases of local transmission.

LL-2024a was selected as the reference strain for *Tm*II* read mapping. The resulting SNP tree (Fig. 4) identified a single cluster of seven isolates with no more than 47 SNPs. Within this cluster, three probable local transmission events were observed, with 0–16 SNPs separating the isolates. Other *Tm*II* isolates exhibited 99–687 SNPs compared to all other isolates and did not appear to be related to any other isolates in this cohort.

## DISCUSSION

### Identification of *Ti*, *Tm*VII, and *Tm*II* isolates

This report presents a comprehensive laboratory investigation of emerging drug-resistant *Ti*, and drug-susceptible *Tm*VII and *Tm* II*, collected from NYC and surrounding counties. Although significant differences exist in infection characteristics and antifungal resistance profiles among these pathogens, accurately identifying isolates at the species and genotype levels remains challenging. Molecular techniques offer high accuracy for species and genotype identification, but their application is often constrained by cost and limited availability. A real-time PCR assay for the identification and detection of *Ti* in isolates and primary specimens was recently reported (36). Furthermore, a comparative study of various dermatophyte diagnostic tests involving several members of the *TiTm*SC demonstrated that while most techniques could identify isolates as *Trichophyton* spp., none could differentiate among *TiTm*SC genotypes except for MALDI-TOF MS identification of *Ti* (2, 5). Although some groups have achieved a 96% identification rate for *Ti* using MALDI-TOF MS, there is currently no commercially available MALDI database containing *Ti* spectra or other *TiTm*SC members at the genotype level.

Currently, ITS sequencing is considered the gold standard for dermatophyte identification due to its availability, specificity, and relatively rapid turnaround time (15, 29). However, manual analysis of *TiTm*SC ITS sequences and accurate assignment of species and genotype can be time-consuming and challenging, particularly because high-quality sequence data spanning the entire ITS region is necessary for precise species and genotype determination. Although a bioinformatic pipeline is available to assist with genotype identification (6), it does not eliminate the labor-intensive process of manually trimming ITS sequences. In this study, a fully automated pipeline was developed to clean, trim, and align Sanger sequencing chromatogram files, extract a consensus sequence of sufficient length when sequence quality permits, and match it to one of the 28 genotypes in the *TiTm*SC. This pipeline removes the need for manual sequence processing and enables simultaneous analysis of sequence data from multiple isolates, thereby accelerating dermatophyte identification, especially during large outbreaks.

### Monitoring for antifungal resistance

Clinical breakpoints for antifungal drugs are currently not available for any dermatophytes. However, we observed that 57% of our NYC *Ti* isolates had moderate to high MICs to the first-line drug, terbinafine, which correlated with mutations L393S and F397L in SQLE. These results are consistent with our earlier report (15), and these findings are not surprising as several studies have reported that *Ti* is increasingly found to be resistant to terbinafine due to amino acid substitution in SQLE at positions L393, F397, F415, and H440 (1, 13, 15, 18, 20, 34, 37–40). Interestingly, and in contrast to a global analysis of *Ti* isolates (16, 41, 42), only some of the NYC terbinafine-resistant isolates showed clustering in the *Ti* phylogenetic tree, further suggesting both local transmission and independent introduction. Elevated MICs against azoles were also observed in some *Ti* isolates, raising concerns over the potential evolution of multidrug-resistant isolates, which would further complicate patient care. Azole resistance has been associated with the overexpression of or mutation in the *CYP51B* gene, which encodes a sterol 14α-demethylase (43). While some SNPs in *CYP51B* were found in our *Ti* isolates, there was no correlation between any SNP(s) and elevated azole MICs (data not shown), which is similar to results reported by others (16, 19). However, we cannot rule out the possibility of *CYP51B* overexpression due to gene duplication, a previously documented resistance mechanism identified via long-read sequencing (16, 43). Other mechanisms of resistance are currently unknown, but overexpression of transporters, other drug efflux pumps, biofilm formations, and loss of mitochondrial complexes have been linked to azole resistance (44, 45).

Currently, only low MICs to terbinafine and azoles have been reported in *Tm*VII or *Tm*II* isolates. Nevertheless, the widespread use of topical combination creams and the empirical treatment of infections prior to organism identification and AFST may promote the development of resistance through mechanisms analogous to those observed in *Ti*. Therefore, AFST should be widely available across healthcare settings to support the optimal selection of antifungal agents, shorten treatment duration, and limit the emergence of resistance.

### Relatedness within *Ti*, *Tm*VII, and *Tm*II* isolates

Whole-genome sequencing was used to assess genetic relatedness within *Ti*, *Tm*VII, and *Tm*II* isolates. In NYC, four major clusters of *Ti* isolates were identified, consistent with recent reports (41, 42). Interpreting these results remains challenging due to the lack of a standardized SNP cutoff for defining relatedness in dermatophyte outbreaks. Sequencing of multiple isolates from the same patient has revealed zero to two SNP differences (16). Previous studies utilizing comprehensive patient epidemiological data identified two probable cases of household transmission in NYC, with zero to two SNPs observed between patient isolates (15). Additional isolates from that study were collected from patients who had recently traveled to Bangladesh or had a family member who traveled there and exhibited 32 to 373 SNP differences from other *Ti* isolates, indicating travel-associated, independent introductions (15). In the current study, an additional pair of NYC *Ti* isolates differing by only three SNPs was identified, likely indicating a local transmission event. However, it remains unclear whether higher SNP differences within each of the four main clusters are attributable to local transmission or to separate travel-related introductions. Our analysis of 17 *Ti* isolates from patients in the same zip code in the present investigation did not show strong clustering. These findings suggest that significant local transmission of *Ti* in NYC is less likely than travel-related introductions. In one global study, a cluster of 22 Chinese *Ti* isolates harboring 7–36 SNPswas attributed to the local spread of a travel-introduced strain. Another study of *Ti* isolates from five hospitals in North India over a 5-year period found only 42 SNPs among all isolates, concluding that these were likely due to clonal spread (37). Two Singaporean *Ti* isolates with 92 SNPs between them, and fewer than 182 SNPs from isolates collected from patients in India, were also considered related (46). More extensive sampling and additional comprehensive studies, including epidemiological patient data with travel history, are required to establish an approximate SNP cutoffs that can differentiate between local transmission events and travel-associated introductions.

Following our initial report of *Tm*VII infections (29), we have observed a rapid increase in *Tm*VII cases in NYC. Unlike *Ti* infections, the increase in *Tm*VII cases in NYC appears to result primarily from clonal spread, as most isolates are clustered in the phylogenetic tree and differed by less than 19 SNPs. The precise mechanisms underlying the spread of *Tm*VII infection are unclear. Current evidence suggests that transmission occurs mainly through direct skin-to-skin contact during sexual activity, pubic hair grooming, or regular use of shared spaces like gyms and saunas (1, 26). These findings highlight the need for further research to elucidate the dynamics of *Tm*VII infection and transmission.

This investigation identified 14 patients infected with *Tm*II*, marking the first documented cases of this pathogen in both NYC and the United States. Patients had either tinea capitis (scalp infection) or tinea corporis (ringworm on the body). The ability of *Tm*II* to infect both the scalp and the body is a characteristic that sets it apart within the taxonomically complex *TiTm*SC (1). Furthermore, female patients outnumbered male patients with *Tm*II* infection, which aligns with a previously published report, although that report included only two cases (1). Whole-genome sequencing of *Tm*II* isolates revealed that half clustered together with fewer than 47 SNPs, suggesting at least a few instances of local transmission with 0–16 SNPs between isolates. The remaining *Tm*II* isolates did not appear closely related and are likely attributed to independent introductions.

### Conclusion

The results presented above highlight the necessity for clinicians to remain vigilant regarding evolving genotypes, atypical clinical presentations, and antifungal resistance to ensure timely diagnosis and appropriate treatment for *Trichophyton* infections. The rapid spread of drug-resistant *Ti*, sexually transmitted *Tm*VII, and newly emerging *Tm*II* further underscores the importance of accurate species and genotype-level identification. Moreover, the increasing incidence of antifungal resistance and treatment failure necessitates enhanced antifungal stewardship, particularly when systemic antifungals are used alone or in combination with antifungals or steroid creams. Both clonal and non-clonal transmission of these pathogens has been documented, demonstrating their capacity for widespread dissemination. A more comprehensive understanding of *Ti, Tm*VII*,* and *Tm*II* transmission pathways is necessary, alongside targeted educational initiatives for clinicians, healthcare professionals, and patients. Effective management should encompass appropriate antifungal selection, application of AFST to determine *in vitro* resistance, elimination of environmental reservoirs, development of clear clinical guidelines, and patient education on household hygiene and textile disinfection to prevent transmission, reinfection, and unnecessary antifungal use.

## AFTER EPUB

*[This article was published on 10 April 2026 with an error in Table 1. The Table 1 was corrected in the current version, posted on 16 April 2026.]*

## SUPPLEMENTAL MATERIAL

File S1 jcm.00156-26-s0001.html Coding for CNN model for Trichophytone species and genotyping.

File S2 jcm.00156-26-s0002.docx Details on modeling and WGS.

Figure S1 jcm.00156-26-s0003.tif Alignment of ITS sequences for the 28 genotypes of the TiTmSC.

Figures S2a-c jcm.00156-26-s0004.docx Pipeline analysis.

Figure S3a jcm.00156-26-s0005.tif Determining the best reference strain. (a) K-mer analysis of a representative from our TiTmSC isolate assemblies along with seven GenBank assemblies. A T. rubrum isolate is included as an outgroup.

Figure S3b jcm.00156-26-s0006.tif Determining the best reference strain. (b) Matrix of the average nucleotide identity (ANI) between GenBank assemblies and representatives from our TiTmSC isolate assemblies. A T. rubrum isolate is included as an outgroup.

Figure S4 jcm.00156-26-s0007.tif Maximum-likelihood SNP tree of Ti isolates from patients living in a single zip code.

Supplemental legends jcm.00156-26-s0008.docx Legends for Figures S1 to S4.

Tables S1 to S4 jcm.00156-26-s0009.xlsx Details of WGS and pipeline verification data.

ASM does not own the copyrights to Supplemental Material that may be linked to, or accessed through, an article. The authors have granted ASM a non-exclusive, world-wide license to publish the Supplemental Material files. Please contact the corresponding author directly for reuse.
