# Supplementary material for: The emergence of superficial dermatophytosis due to Trichophyton indotineae and Trichophyton mentagrophytes genotypes VII and II* in New York: a need for comprehensive testing approaches
Source: J Clin Microbiol. 2026 Apr 10;64(5):e00156-26. doi: 10.1128/jcm.00156-26 (PMC13170464; doi:10.1128/jcm.00156-26)
Supplement: File S1 — Coding for CNN model for Trichophytone species and genotyping. [file jcm.00156-26-s0001.html]

Modeling with OOD


# Convolutional Neural Network (CNN) model for *Trichophyton mentagrophytes*/*T. interdigitale* species complex (*TiTm*SC) Genotype Classification using K-mer Features¶

The internal transcribed spacer (ITS) region in fungal pathogens is a variable sequence located between the conserved 18S and 28S rDNA genes and serves as a unique barcode for each fungal species. It consists of a variable ITS1 region, the conserved 5.8S region, and a variable ITS2 region. The typical length of the ITS sequence is between 300-800 base pairs. Based on the ITS sequence, a fungal pathogen can be identified to the Genus, species, and even genotype level.

There are 28 members of the *Trichophyton mentagrophytes*/*T. interdigitale* species complex (*TiTm*SC). These pathogenic fungi are dermatophytes that cause infections such as tinea pedis (athlete’s foot) and onychomycosis (nail infections), as well as highly inflammatory lesions on the scalp and body (ringworm). The location of infection, severity, mechanism of spread, and resistance to antifungal treatments vary depending on the species and genotype of the infectious agent. Distinguishing among the 28 members of the *TiTm*SC is notoriously difficult and requires sequencing and analysis of the ITS region.

Manual analysis of ITS sequences can be cumbersome and time-consuming. To aid in the rapid identification of *TiTm*SC pathogens, we have developed an automated pipeline that takes raw ITS Sanger sequencing files (.ab1) as input and will accurately output the identity of the pathogenic fungi to the genotype level using a convolutional neural network (CNN) trained on the k-mer frequency of reference ITS sequences. The pipeline returns the predicted genotype, the confidence score, and the Mahalanobis distance. We have validated our model on 382 manually analyzed ITS sequences from patient samples, and it accurately classified 98.7% (the remaining 1.3% required manual analysis due to high background in the chromatograms). Users can also input a manually analyzed ITS sequence and obtain a *TiTm*SC genotype/or run multiple sequences simultaneously. These features enable rapid, accurate genotyping of *TiTm*SC isolates from ITS Sanger sequencing.

In [145]:

```
## Libraries
import pandas as pd
import numpy as np
import matplotlib.pyplot as plt
import torch
from Bio import SeqIO, AlignIO
from collections import Counter
from sklearn.preprocessing import StandardScaler
from sklearn.feature_extraction.text import CountVectorizer
import tensorflow as tf
import warnings
warnings.filterwarnings("ignore")
```

## Sanger Sequencing Pipeline¶

In [146]:

```
import re
import subprocess
import os
import io
from functools import partial
from io import BytesIO, StringIO
from Bio import SeqIO
from Bio.Align import PairwiseAligner
from Bio.Seq import Seq
from Bio.SeqRecord import SeqRecord
import pandas as pd
import streamlit as st
from concurrent.futures import ThreadPoolExecutor, as_completed

#Loading raw chromatograms
def load_abi_file(file_io):
    record = SeqIO.read(file_io, "abi")
    return str(record.seq), record.letter_annotations["phred_quality"]

#Trimming low quality bases
def trim_ends(sequence, quality_scores, window_size, quality_threshold, min_length):
    L = len(sequence)
    # left bound
    left = 0
    for i in range(L - window_size + 1):
        if all(q >= quality_threshold for q in quality_scores[i: i + window_size]):
            left = i
            break
    # right bound
    right = L
    for i in range(L - window_size, -1, -1):
        if all(q >= quality_threshold for q in quality_scores[i: i + window_size]):
            right = i + window_size
            break
    trimmed_seq = sequence[left:right]
    trimmed_qual = quality_scores[left:right]
    if len(trimmed_seq) < min_length:
        raise ValueError(f"Trimmed length {len(trimmed_seq)} < min_length {min_length}")
    return trimmed_seq, trimmed_qual

# reverse complementing the sequence
def reverse_complement(sequence, quality_scores):
    rev_seq = str(Seq(sequence).reverse_complement())
    rev_qual = quality_scores[::-1]
    return rev_seq, rev_qual

# trimming extra sequence after alignment
def trim_overhangs(aln, fwd_seq, fwd_qual, rev_seq, rev_qual):


    fwd_segments = aln.aligned[0]
    rev_segments = aln.aligned[1]


    fwd_start = fwd_segments[0][0]
    fwd_end = fwd_segments[-1][1]
    rev_start = rev_segments[0][0]
    rev_end = rev_segments[-1][1]

    aligned_fwd = str(aln[0])
    aligned_rev = str(aln[1])

    fwd_qual_trimmed = fwd_qual[fwd_start:fwd_end]
    rev_qual_trimmed = rev_qual[rev_start:rev_end]

    f_idx = 0
    r_idx = 0
    aligned_fwd_qual = []
    aligned_rev_qual = []

    for base in aligned_fwd:
        if base == '-':
            aligned_fwd_qual.append(0)
        else:
            aligned_fwd_qual.append(fwd_qual_trimmed[f_idx])
            f_idx += 1

    for base in aligned_rev:
        if base == '-':
            aligned_rev_qual.append(0)
        else:
            aligned_rev_qual.append(rev_qual_trimmed[r_idx])
            r_idx += 1

    return aligned_fwd, aligned_fwd_qual, aligned_rev, aligned_rev_qual

# building  consensus sequence
def build_consensus(seq1, qual1, seq2, qual2, min_quality):


    cons = []
    for b1, b2, q1, q2 in zip(seq1, seq2, qual1, qual2):

        if b1 == '-' and b2 == '-':
            cons.append('-')
        elif b1 == '-':
            cons.append(b2 if q2 >= min_quality else 'N')
        elif b2 == '-':
            cons.append(b1 if q1 >= min_quality else 'N')

        elif b1 == b2:

            if q1 >= min_quality or q2 >= min_quality:
                cons.append(b1)
            else:
                cons.append('N')

        elif q1 > q2:
            cons.append(b1 if q1 >= min_quality else 'N')
        elif q2 > q1:
            cons.append(b2 if q2 >= min_quality else 'N')
        else:

            if b1 != 'N' and q1 >= min_quality:
                cons.append(b1)
            elif b2 != 'N' and q2 >= min_quality:
                cons.append(b2)
            else:
                cons.append('N')

    return "".join(cons)

def remove_n_runs(sequence):
    return re.sub(r"N+", "N", sequence)

def extract_longest_n_free_subseq(sequence):
    segments = sequence.split("N")
    return max(segments, key=len) if segments else ""

def write_fasta(sequence, name="consensus"):
    rec = SeqRecord(Seq(sequence), id=name, description=f"{len(sequence)} bp")
    text_buf = StringIO()
    SeqIO.write(rec, text_buf, "fasta")
    fasta_str = text_buf.getvalue()
    byte_buf = BytesIO(fasta_str.encode("utf-8"))
    byte_buf.seek(0)
    return byte_buf

# Taking forward and reverse files aligning, trimming and QC to get  consensus sequence
def ab1_processing_pipeline(forward_file, reverse_file, window_size=5, quality_threshold=8, min_length=5, min_quality=5):


    f_seq, f_qual = load_abi_file(forward_file)
    r_seq, r_qual = load_abi_file(reverse_file)


    f_seq, f_qual = trim_ends(f_seq, f_qual, window_size, quality_threshold, min_length)
    r_seq, r_qual = trim_ends(r_seq, r_qual, window_size, quality_threshold, min_length)


    r_seq, r_qual = reverse_complement(r_seq, r_qual)


    aligner = PairwiseAligner()
    aligner.mode = "local"
    aligner.match_score = 2.0
    aligner.mismatch_score = -1.0
    aligner.open_gap_score = -3.0
    aligner.extend_gap_score = -1.0
    aln = aligner.align(r_seq, f_seq)[0]


    r_segments = aln.aligned[0]
    f_segments = aln.aligned[1]

    r_start = r_segments[0][0]
    r_end = r_segments[-1][1]
    f_start = f_segments[0][0]
    f_end = f_segments[-1][1]


    #consensus sequence
    left_overhang = r_seq[:r_start]
    left_overhang_qual = r_qual[:r_start]

    #Aligned region
    aligned_r = str(aln[0])
    aligned_f = str(aln[1])
    r_qual_trimmed = r_qual[r_start:r_end]
    f_qual_trimmed = f_qual[f_start:f_end]

    # Map quality scores to aligned positions
    r_idx = 0
    f_idx = 0
    aligned_r_qual = []
    aligned_f_qual = []

    for base in aligned_r:
        if base == '-':
            aligned_r_qual.append(0)
        else:
            aligned_r_qual.append(r_qual_trimmed[r_idx])
            r_idx += 1

    for base in aligned_f:
        if base == '-':
            aligned_f_qual.append(0)
        else:
            aligned_f_qual.append(f_qual_trimmed[f_idx])
            f_idx += 1

    # Right overhang from reverse read
    right_overhang = r_seq[r_end:]
    right_overhang_qual = r_qual[r_end:]

    # Build consensus for aligned region
    aligned_cons = build_consensus(aligned_r, aligned_r_qual, aligned_f, aligned_f_qual, min_quality)

    # Add left overhang
    left_cons = ''.join([base if qual >= min_quality else 'N'
                         for base, qual in zip(left_overhang, left_overhang_qual)])

    # Add right overhang
    right_cons = ''.join([base if qual >= min_quality else 'N'
                          for base, qual in zip(right_overhang, right_overhang_qual)])

    # Combine all parts
    full_cons = left_cons + aligned_cons + right_cons


    #  full consensus seqeunce
    full_cons = full_cons.replace('-', '')
    final = extract_longest_n_free_subseq(remove_n_runs(full_cons))

    return final

#Best Param window_size=5, quality_threshold=8, min_length=5, min_quality=5

#Reading all raw ab1 files generating consensus sequence and storing into df
def process_ab1_files(input_dir):
    file_pairs = {}
    for filename in os.listdir(input_dir):
        if filename.endswith(".ab1"):
            parts = filename.split("_")
            name = parts[0]
            version = "_".join(parts[1:]).replace(".ab1", "").lower()
            if name not in file_pairs:
                file_pairs[name] = {"V50": None, "V1827": None, "V49": None}
            if "v50" in version:
                file_pairs[name]["V50"] = os.path.join(input_dir, filename)
            elif "v1827" in version:
                file_pairs[name]["V1827"] = os.path.join(input_dir, filename)
            elif "v49" in version:
                file_pairs[name]["V49"] = os.path.join(input_dir, filename)
    results = []
    for name, files in file_pairs.items():
        forward_file = files.get("V1827")
        reverse_file = files.get("V50")
        v49_file = files.get("V49")
        if forward_file and reverse_file:
            seq_v1827_v50 = ab1_processing_pipeline(forward_file, reverse_file)
            if len(seq_v1827_v50) > 400:
                results.append({
                    "name": name,
                    "sequence": seq_v1827_v50,
                })
            else:
                if v49_file:
                    seq_v49_v50 = ab1_processing_pipeline(v49_file, reverse_file)
                    if len(seq_v49_v50) < 200:
                        results.append({
                            "name": name,
                            "sequence": seq_v49_v50,
                        })
                    else:
                        results.append({
                            "name": name,
                            "sequence": seq_v49_v50,
                        })
                else:
                    results.append({
                        "name": name,
                        "sequence": seq_v1827_v50,
                    })
        else:
            results.append({
                "name": name,
                "sequence": "",
            })
    df = pd.DataFrame(results)
    df['Query_Sequence_length'] = df['sequence'].str.len()
    return df

# whole pipeline from sanger sequence raw files to DataFrame with file name and its consensus sequence
patient_seq = process_ab1_files(r'sanger_files')
patient_seq
```

Out[146]:

|  | name | sequence | Query\_Sequence\_length |
| --- | --- | --- | --- |
| 0 | patientAA | AACAAGGTTTCCGTAGGTGAACCTGCGGAAGGATCATTAACGCGCA... | 646 |
| 1 | patientAB | ACAAGGTTTCCGTAGGTGAACCTGCGGAAGGATCATTAACGCGCAG... | 644 |
| 2 | patientAC | AACAAGGTTTCCGTAGGTGAACCTGCGGAAGGATCATTAACGCGCA... | 641 |
| 3 | patientAD | AACAAGGTTTCCGTAGGTGAACCTGCGGAAGGATCATTAACGCGCA... | 641 |
| 4 | patientAE | AACAAGGTTTCCGTAGGTGAACCTGCGGAAGGATCATTAACGCGCA... | 649 |
| 5 | patientAF | AACAAGGTTTCCGTAGGTGAACCTGCGGAAGGATCATTAACGCGCA... | 641 |
| 6 | patientAG | GTCGTAACAAGGTTTCCGTAGGTGAACCTGCGGAAGGATCATTAAC... | 650 |
| 7 | patientAH | ACAAGGTTTCCGTAGGTGAACCTGCGGAAGGATCATTAACGCGCAG... | 642 |
| 8 | patientAI | TAACAAGGTTTCCGTAGGTGAACCTGCGGAAGGATCATTAACGCGC... | 641 |
| 9 | patientAJ | TAACAAGGTTTCCGTAGGTGAACCTGCGGAAGGATCATTAACGCGC... | 642 |
| 10 | patientAK | TAACAAGGTTTCCGTAGGTGAACCTGCGGAAGGATCATTAACGCGC... | 643 |
| 11 | patientAL | TAACAAGGTTTCCGTAGGTGAACCTGCGGAAGGATCATTAACGCGC... | 642 |
| 12 | patientAM | TAACAAGGTTTCCGTAGGTGAACCTGCGGAAGGATCATTAACGCGC... | 643 |
| 13 | patientAN | AGTAAAAAGTCGTAACAAGGTTTCCGTAGGTGAACCTGCGGAAGGA... | 656 |
| 14 | patientAO | AACAAGGTTTCCGTAGGTGAACCTGCGGAAGGATCATTAACGCGCA... | 641 |
| 15 | patientAP | GTCGTAACAAGGTTTCCGTAGGTGAACCTGCGGAAGGATCATTAAC... | 654 |
| 16 | patientAQ | GTAACAAGGTTTCCGTAGGTGAACCTGCGGAAGGATCATTAGCGCG... | 651 |
| 17 | patientAR | GTAACAAGGTTTCCGTAGGTGAACCTGCGGAAGGATCATTAGCGCG... | 645 |
| 18 | patientAS | AACAAGGTTTCCGTAGGTGAACCTGCGGAAGGATCATTAACGCGCA... | 648 |
| 19 | patientAT | TAACAAGGTTTCCGTAGGTGAACCTGCGGAAGGATCATTAACGCGC... | 644 |
| 20 | patientAU | TCGTAACAAGGTTTCCGTAGGTGAACCTGCGGAAGGATCATTAACG... | 647 |
| 21 | patientA | GCGGAAGGATCATTAGCGCGCAGGCCGGAGGCTGGCCCCCCACGAT... | 619 |
| 22 | PatientBA | TGATTCAAAACAAAAACAAGTCAAAACTTTTAACAACGGATCTCTT... | 362 |
| 23 | PatientBB | TAACAAGGTTTCCGTAGGTGAACCTGCGGAAGGATCATTAACGCGC... | 660 |
| 24 | patientBC | TAACAAGGTTTCCGTAGGTGAACCTGCGGAAGGATCATTACCGAGT... | 548 |

## We are loading all 28 Genotypes which belongs to T. interdigitale/ T. mentagrophytes species complex¶

In [117]:

```
## Loading Reference Data

def clean_label(label):
    label_parts = label.split('_')[1:]
    return ' '.join(label_parts)

fasta_path = "Ti-Tm species complex alignment ordered trimmed.fa"
genotypes, sequences = [], []

for record in SeqIO.parse(fasta_path, "fasta"):
    genotypes.append(record.id)
    sequences.append(str(record.seq))

df = pd.DataFrame({"Genotype": genotypes, "Sequence": sequences})

df['Genotype'] = df['Genotype'].apply(clean_label)
df['Sequence'] = df['Sequence']
df['Sequence_length'] = df['Sequence'].str.len()
df
```

Out[117]:

|  | Genotype | Sequence | Sequence\_length |
| --- | --- | --- | --- |
| 0 | Trichophyton interdigitale I | GCGCGCAGGCCGGAGGCTGGCCCCCCACGATAGGGCCAAACGTCCG... | 598 |
| 1 | Trichophyton interdigitale II | GCGCGCAGGCCGGAGGCTGGCCCCCCACGATAGGGCCAAACGTCCG... | 598 |
| 2 | Trichophyton mentagrophytes II\* | ACGCGCAGGCCGGAGGCTGGCCCCCCACGATAGGGCCAAACGTCCG... | 598 |
| 3 | Trichophyton mentagrophytes III | ACGCGCAGGCCGGAGGCTGGCCCCCCACGATAGGGCCAAACGTCCG... | 598 |
| 4 | Trichophyton mentagrophytes III\* | ACGCGCAGGCCGGAGGCTGGCCCCCCACGATAGGGCCAAACGTCCG... | 598 |
| 5 | Trichophyton mentagrophytes IV | ACGCGCAGGCCGGACGCTGGCCCCCCACGATAGGGCCAAACGTCCG... | 598 |
| 6 | Trichophyton mentagrophytes V | ACGCGCAGGCCGGAGGCTGGCCCCCCACGATAGGGCCAAACGTCCG... | 598 |
| 7 | Trichophyton mentagrophytes VI | ACGCGCAGGCCGGAGGCTGGCCCCCCACGATAGGGCCAAACGTCCG... | 598 |
| 8 | Trichophyton mentagrophytes VII | ACGCGCAGGCCGGAGGCTGGCCGCCCACGATAGGGCCAAACGTCCG... | 598 |
| 9 | Trichophyton indotineae | ACGCGCAGGCCGGAGGCTGGCCCCCCACGATAGGGCCAAACGTCCG... | 598 |
| 10 | Trichophyton mentagrophytes IX | ACGCGCAGGCCGGAGGCTGGCCCCCCACGATAGGGCCAAACGTCCG... | 598 |
| 11 | Trichophyton interdigitale X | GCGCGCAGGCCGGAGGCTGGCCCCCCACGATAGGGCCAAACGTCCG... | 598 |
| 12 | Trichophyton interdigitale XI | GCGCGCAGGCCGGAGGCTGGCCCCCCACGATAGGGCCAAACGTCCG... | 598 |
| 13 | Trichophyton interdigitale XII | GCGCGCAGGCCGGAGGCTGGCCCCCCACGATAGGGCCAAACGTCCG... | 598 |
| 14 | Trichophyton mentagrophytes XIII | ACGCGCAGGCCGGAGGCTGGCCCCCCACGATAGGGCCAAACGTCCG... | 598 |
| 15 | Trichophyton mentagrophytes XIV | ACGCGCAGGCCGGAGGCTGGCCCCCCACGATAGGGCCAAACGTCCG... | 598 |
| 16 | Trichophyton mentagrophytes XV | ACGCGCAGGCCGGAGGCTGGCCCCCCACGATAGGGCCAAACGTCCG... | 598 |
| 17 | Trichophyton mentagrophytes XVI | ACGCGCAGGCCGGAGGCTGGCCCCCCACGATAGGGCCAAACGTCCG... | 598 |
| 18 | Trichophyton mentagrophytes XVII | ACGCGCAGGCCGGAGGCTGGCCCCCCACGATAGGGCCAAACGTCCG... | 598 |
| 19 | Trichophyton mentagrophytes XVIII | ACGCGCAGGCCGGAGGCTGGCCCCCCACGATAGGGCCAAACGTCCG... | 598 |
| 20 | Trichophyton mentagrophytes XIX | ACGCGCAGGCCGGAGGCTGGCCCCCCACGATAGGGCCAAACGTCCG... | 598 |
| 21 | Trichophyton mentagrophytes XX | ACGCGCAGGCCGGAGGCTGGCCCCCCACGATAGGGCCAAACGTCCG... | 598 |
| 22 | Trichophyton mentagrophytes XXI | ACGCGCAGGCCGGAGGCTGGCCCCCCACGATAGGGCCAAACGTCCG... | 598 |
| 23 | Trichophyton mentagrophytes XXII | ACGCGCAGGCCGGAGGCTGGCCCCCCACGATAGGGCCAAACGTCCG... | 598 |
| 24 | Trichophyton mentagrophytes XXIII | ACGCGCAGGCCGGAGGCTGGCCCCCCACGATAGGGCCAAACGTCCG... | 598 |
| 25 | Trichophyton mentagrophytes XXIV | ACGCGCAGGCCGGAGGCTGGCCCCCCACGATAGGGCCAAACGTCCG... | 598 |
| 26 | interdigitale XXV | GCGCGCAGGCCGGAGGCTGGCCCCCCACGATAGGGCCAAACGTCCG... | 598 |
| 27 | Trichophyton mentagrophytes XXVI | ACGCGCAGGCCGGAGGCTGGCCCCCCACGATAGGGCCAAACGTCCG... | 598 |

In [118]:

```
## Finding out the unique characters in the sequence
chars = sorted(list(set(''.join(df.Sequence))))
print(f'The chars present in the reference sequence are :{chars}')
```

```
The chars present in the reference sequence are :['-', 'A', 'C', 'G', 'T']
```

# Removing '-' from the reference sequence¶

## '-' is the insertion(Gap) in the reference sequence which generated due to alignment. Dropping the '-' character.¶

In [119]:

```
fasta_path = "Ti-Tm species complex alignment ordered trimmed.fa"
genotypes, sequences = [], []

for record in SeqIO.parse(fasta_path, "fasta"):
    genotypes.append(record.id)
    sequences.append(str(record.seq).replace("-", ""))

df = pd.DataFrame({"Genotype": genotypes, "Sequence": sequences})

df['Genotype'] = df['Genotype'].apply(clean_label)
df['Sequence_length'] = df['Sequence'].str.len()
chars = sorted(list(set(''.join(df.Sequence))))


print(f'The chars present in the reference sequence are :{chars}')
```

```
The chars present in the reference sequence are :['A', 'C', 'G', 'T']
```

In [120]:

```
print("the minimum and maximum length of sequence data are :", (min(len(w) for w in df.Sequence), max(len(w) for w in df.Sequence)), "bps")
```

```
the minimum and maximum length of sequence data are : (593, 596) bps
```

## Data Analysis using Kmer features¶

In [121]:

```
from collections import Counter

k = 6
kmer_list = []
genotype_list = []

pair_counter = Counter()

for sequences in df.Sequence:
    genotype = df[df['Sequence'] == sequences].Genotype.item()
    kmers = [sequences[i: i+k] for i in range(len(sequences)- k + 1)]
    for kmer in kmers:
        pair_counter[(genotype, kmer)] += 1
    kmer_list.append(kmers)
    genotype_list.append(genotype)

kmer_dist = list(zip(genotype_list, kmer_list))
count_df = pd.DataFrame([(g, k, c) for (g, k), c in pair_counter.items()],columns=["Genotype", "k-mer", "Count"]).sort_values(by="Count", ascending=False)
count_df = count_df.pivot(index='Genotype', columns='k-mer', values='Count').fillna(0).astype(int)
count_df
```

Out[121]:

| k-mer | AAAAAA | AAAAAT | AAAACT | AAAAGC | AAAATC | AAAATT | AAACCA | AAACGC | AAACGT | AAACTT | ... | TTTATA | TTTCAA | TTTCAG | TTTCTA | TTTGAA | TTTGGA | TTTGGG | TTTTAT | TTTTGG | TTTTTG |
| --- | --- | --- | --- | --- | --- | --- | --- | --- | --- | --- | --- | --- | --- | --- | --- | --- | --- | --- | --- | --- | --- |
| Genotype |  |  |  |  |  |  |  |  |  |  |  |  |  |  |  |  |  |  |  |  |  |
| Trichophyton indotineae | 2 | 2 | 1 | 1 | 2 | 1 | 1 | 1 | 1 | 1 | ... | 1 | 1 | 2 | 0 | 1 | 0 | 1 | 1 | 1 | 1 |
| Trichophyton interdigitale I | 2 | 2 | 1 | 1 | 2 | 1 | 1 | 1 | 1 | 1 | ... | 1 | 1 | 2 | 0 | 1 | 0 | 1 | 1 | 0 | 0 |
| Trichophyton interdigitale II | 2 | 2 | 1 | 1 | 2 | 1 | 1 | 1 | 1 | 1 | ... | 1 | 1 | 2 | 0 | 1 | 0 | 1 | 1 | 0 | 0 |
| Trichophyton interdigitale X | 2 | 2 | 1 | 1 | 2 | 1 | 1 | 1 | 1 | 1 | ... | 1 | 1 | 2 | 1 | 1 | 0 | 1 | 1 | 0 | 0 |
| Trichophyton interdigitale XI | 2 | 2 | 1 | 1 | 2 | 1 | 1 | 1 | 1 | 1 | ... | 1 | 1 | 2 | 0 | 1 | 0 | 1 | 1 | 0 | 0 |
| Trichophyton interdigitale XII | 2 | 2 | 1 | 1 | 2 | 1 | 1 | 1 | 1 | 1 | ... | 1 | 1 | 2 | 0 | 1 | 0 | 1 | 1 | 0 | 0 |
| Trichophyton mentagrophytes II\* | 2 | 2 | 1 | 1 | 2 | 1 | 1 | 1 | 1 | 1 | ... | 1 | 1 | 2 | 0 | 1 | 0 | 1 | 1 | 0 | 0 |
| Trichophyton mentagrophytes III | 2 | 2 | 1 | 1 | 2 | 1 | 1 | 1 | 1 | 1 | ... | 1 | 1 | 2 | 0 | 1 | 0 | 1 | 1 | 0 | 0 |
| Trichophyton mentagrophytes III\* | 2 | 2 | 1 | 1 | 2 | 1 | 1 | 1 | 1 | 1 | ... | 1 | 1 | 2 | 0 | 1 | 0 | 1 | 1 | 0 | 0 |
| Trichophyton mentagrophytes IV | 3 | 2 | 1 | 1 | 2 | 1 | 1 | 0 | 1 | 1 | ... | 1 | 1 | 2 | 0 | 1 | 0 | 1 | 1 | 0 | 0 |
| Trichophyton mentagrophytes IX | 2 | 2 | 1 | 1 | 2 | 1 | 1 | 0 | 1 | 1 | ... | 1 | 1 | 2 | 0 | 1 | 1 | 0 | 1 | 0 | 0 |
| Trichophyton mentagrophytes V | 2 | 2 | 1 | 1 | 2 | 1 | 1 | 0 | 1 | 1 | ... | 1 | 1 | 2 | 0 | 1 | 0 | 1 | 1 | 0 | 0 |
| Trichophyton mentagrophytes VI | 2 | 2 | 1 | 1 | 2 | 1 | 1 | 0 | 1 | 1 | ... | 1 | 1 | 2 | 0 | 1 | 0 | 1 | 1 | 0 | 0 |
| Trichophyton mentagrophytes VII | 2 | 2 | 1 | 1 | 2 | 1 | 1 | 0 | 1 | 1 | ... | 1 | 1 | 2 | 0 | 1 | 0 | 1 | 1 | 0 | 0 |
| Trichophyton mentagrophytes XIII | 2 | 2 | 1 | 1 | 2 | 1 | 1 | 1 | 1 | 1 | ... | 1 | 1 | 2 | 0 | 1 | 0 | 1 | 1 | 1 | 1 |
| Trichophyton mentagrophytes XIV | 2 | 2 | 1 | 1 | 2 | 1 | 1 | 1 | 1 | 1 | ... | 1 | 1 | 2 | 0 | 1 | 0 | 1 | 1 | 1 | 1 |
| Trichophyton mentagrophytes XIX | 2 | 2 | 1 | 1 | 2 | 1 | 1 | 1 | 1 | 1 | ... | 1 | 1 | 2 | 0 | 1 | 0 | 1 | 1 | 0 | 0 |
| Trichophyton mentagrophytes XV | 2 | 2 | 1 | 1 | 2 | 1 | 1 | 1 | 1 | 1 | ... | 1 | 1 | 2 | 0 | 1 | 0 | 1 | 1 | 0 | 0 |
| Trichophyton mentagrophytes XVI | 2 | 2 | 1 | 1 | 2 | 1 | 1 | 1 | 1 | 1 | ... | 1 | 1 | 2 | 0 | 1 | 0 | 1 | 1 | 0 | 0 |
| Trichophyton mentagrophytes XVII | 2 | 2 | 1 | 1 | 2 | 1 | 1 | 1 | 1 | 1 | ... | 1 | 1 | 2 | 0 | 1 | 0 | 1 | 1 | 0 | 0 |
| Trichophyton mentagrophytes XVIII | 2 | 2 | 1 | 1 | 2 | 1 | 1 | 1 | 1 | 1 | ... | 1 | 1 | 2 | 0 | 1 | 0 | 1 | 1 | 0 | 0 |
| Trichophyton mentagrophytes XX | 3 | 2 | 1 | 1 | 2 | 1 | 1 | 0 | 1 | 1 | ... | 1 | 1 | 2 | 0 | 1 | 0 | 1 | 1 | 0 | 0 |
| Trichophyton mentagrophytes XXI | 2 | 2 | 1 | 1 | 2 | 1 | 1 | 0 | 1 | 1 | ... | 1 | 1 | 2 | 0 | 1 | 0 | 1 | 1 | 0 | 0 |
| Trichophyton mentagrophytes XXII | 2 | 2 | 1 | 1 | 2 | 1 | 1 | 0 | 1 | 1 | ... | 1 | 1 | 2 | 0 | 1 | 0 | 1 | 1 | 0 | 0 |
| Trichophyton mentagrophytes XXIII | 2 | 2 | 1 | 1 | 1 | 1 | 1 | 0 | 1 | 1 | ... | 1 | 1 | 2 | 0 | 1 | 0 | 1 | 1 | 0 | 0 |
| Trichophyton mentagrophytes XXIV | 2 | 2 | 1 | 1 | 2 | 1 | 1 | 0 | 1 | 1 | ... | 1 | 1 | 2 | 0 | 1 | 0 | 1 | 1 | 0 | 0 |
| Trichophyton mentagrophytes XXVI | 2 | 2 | 1 | 1 | 2 | 1 | 1 | 1 | 1 | 1 | ... | 1 | 1 | 2 | 0 | 1 | 0 | 1 | 1 | 0 | 0 |
| interdigitale XXV | 2 | 2 | 1 | 1 | 2 | 1 | 1 | 1 | 1 | 1 | ... | 1 | 1 | 2 | 0 | 1 | 0 | 1 | 1 | 0 | 0 |

28 rows × 683 columns

## Finding most repeated Kmers throughout all sequence's which are called as Motifs(motif is a short repeating sequence)¶

In [122]:

```
## Finding the Motifs(motif is a short repeating sequence)
k = 6
motif_count = Counter()
for sequences in df.Sequence:
    for i in range(len(sequences) - k +1):
        kmer = sequences[i:i+k]
        motif_count[kmer] +=1

kmer_list = motif_count.most_common()
motif_df = pd.DataFrame(kmer_list, columns=["kmer", "count"])
motif_df
```

Out[122]:

|  | kmer | count |
| --- | --- | --- |
| 0 | GCGCCC | 112 |
| 1 | GCCCCC | 88 |
| 2 | GGCCGC | 85 |
| 3 | TTCCGG | 84 |
| 4 | ATTCCG | 84 |
| ... | ... | ... |
| 678 | CCCTCT | 1 |
| 679 | CCTCTG | 1 |
| 680 | CTCTGG | 1 |
| 681 | TCTGGC | 1 |
| 682 | TTATTA | 1 |

683 rows × 2 columns

In [123]:

```
import matplotlib.pyplot as plt
import seaborn as sns
# Plot
plt.figure(figsize=(10, 6))
sns.barplot(data=motif_df[:10], x='kmer', y='count', palette='gray')
plt.title('Top 10 Most Frequent 6-mers')
plt.xlabel('6-mer')
plt.ylabel('Count')
plt.tight_layout()
plt.show()
```

## Model Building¶

In [124]:

```
fasta_path = "Ti-Tm species complex alignment ordered trimmed.fa"
genotypes, sequences = [], []

for record in SeqIO.parse(fasta_path, "fasta"):
    genotypes.append(record.id)
    sequences.append(str(record.seq).replace("-", ""))

df = pd.DataFrame({"Genotype": genotypes, "Sequence": sequences})
df['Sequence_length'] = df['Sequence'].str.len()
df['Genotype'] = df['Genotype'].apply(clean_label)
df
```

Out[124]:

|  | Genotype | Sequence | Sequence\_length |
| --- | --- | --- | --- |
| 0 | Trichophyton interdigitale I | GCGCGCAGGCCGGAGGCTGGCCCCCCACGATAGGGCCAAACGTCCG... | 594 |
| 1 | Trichophyton interdigitale II | GCGCGCAGGCCGGAGGCTGGCCCCCCACGATAGGGCCAAACGTCCG... | 593 |
| 2 | Trichophyton mentagrophytes II\* | ACGCGCAGGCCGGAGGCTGGCCCCCCACGATAGGGCCAAACGTCCG... | 593 |
| 3 | Trichophyton mentagrophytes III | ACGCGCAGGCCGGAGGCTGGCCCCCCACGATAGGGCCAAACGTCCG... | 593 |
| 4 | Trichophyton mentagrophytes III\* | ACGCGCAGGCCGGAGGCTGGCCCCCCACGATAGGGCCAAACGTCCG... | 593 |
| 5 | Trichophyton mentagrophytes IV | ACGCGCAGGCCGGACGCTGGCCCCCCACGATAGGGCCAAACGTCCG... | 594 |
| 6 | Trichophyton mentagrophytes V | ACGCGCAGGCCGGAGGCTGGCCCCCCACGATAGGGCCAAACGTCCG... | 594 |
| 7 | Trichophyton mentagrophytes VI | ACGCGCAGGCCGGAGGCTGGCCCCCCACGATAGGGCCAAACGTCCG... | 593 |
| 8 | Trichophyton mentagrophytes VII | ACGCGCAGGCCGGAGGCTGGCCGCCCACGATAGGGCCAAACGTCCG... | 593 |
| 9 | Trichophyton indotineae | ACGCGCAGGCCGGAGGCTGGCCCCCCACGATAGGGCCAAACGTCCG... | 593 |
| 10 | Trichophyton mentagrophytes IX | ACGCGCAGGCCGGAGGCTGGCCCCCCACGATAGGGCCAAACGTCCG... | 593 |
| 11 | Trichophyton interdigitale X | GCGCGCAGGCCGGAGGCTGGCCCCCCACGATAGGGCCAAACGTCCG... | 593 |
| 12 | Trichophyton interdigitale XI | GCGCGCAGGCCGGAGGCTGGCCCCCCACGATAGGGCCAAACGTCCG... | 593 |
| 13 | Trichophyton interdigitale XII | GCGCGCAGGCCGGAGGCTGGCCCCCCACGATAGGGCCAAACGTCCG... | 593 |
| 14 | Trichophyton mentagrophytes XIII | ACGCGCAGGCCGGAGGCTGGCCCCCCACGATAGGGCCAAACGTCCG... | 593 |
| 15 | Trichophyton mentagrophytes XIV | ACGCGCAGGCCGGAGGCTGGCCCCCCACGATAGGGCCAAACGTCCG... | 593 |
| 16 | Trichophyton mentagrophytes XV | ACGCGCAGGCCGGAGGCTGGCCCCCCACGATAGGGCCAAACGTCCG... | 593 |
| 17 | Trichophyton mentagrophytes XVI | ACGCGCAGGCCGGAGGCTGGCCCCCCACGATAGGGCCAAACGTCCG... | 593 |
| 18 | Trichophyton mentagrophytes XVII | ACGCGCAGGCCGGAGGCTGGCCCCCCACGATAGGGCCAAACGTCCG... | 593 |
| 19 | Trichophyton mentagrophytes XVIII | ACGCGCAGGCCGGAGGCTGGCCCCCCACGATAGGGCCAAACGTCCG... | 593 |
| 20 | Trichophyton mentagrophytes XIX | ACGCGCAGGCCGGAGGCTGGCCCCCCACGATAGGGCCAAACGTCCG... | 594 |
| 21 | Trichophyton mentagrophytes XX | ACGCGCAGGCCGGAGGCTGGCCCCCCACGATAGGGCCAAACGTCCG... | 595 |
| 22 | Trichophyton mentagrophytes XXI | ACGCGCAGGCCGGAGGCTGGCCCCCCACGATAGGGCCAAACGTCCG... | 596 |
| 23 | Trichophyton mentagrophytes XXII | ACGCGCAGGCCGGAGGCTGGCCCCCCACGATAGGGCCAAACGTCCG... | 594 |
| 24 | Trichophyton mentagrophytes XXIII | ACGCGCAGGCCGGAGGCTGGCCCCCCACGATAGGGCCAAACGTCCG... | 594 |
| 25 | Trichophyton mentagrophytes XXIV | ACGCGCAGGCCGGAGGCTGGCCCCCCACGATAGGGCCAAACGTCCG... | 593 |
| 26 | interdigitale XXV | GCGCGCAGGCCGGAGGCTGGCCCCCCACGATAGGGCCAAACGTCCG... | 593 |
| 27 | Trichophyton mentagrophytes XXVI | ACGCGCAGGCCGGAGGCTGGCCCCCCACGATAGGGCCAAACGTCCG... | 593 |

In [125]:

```
from Bio import SeqIO
import pandas as pd
import re

fasta_path = "Ti-Tm species complex alignment ordered trimmed.fa"
genotypes, sequences = [], []

for record in SeqIO.parse(fasta_path, "fasta"):
    genotypes.append(record.id)
    sequences.append(str(record.seq).replace("-", ""))

validation_df = pd.DataFrame({"Genotype": genotypes, "Sequence": sequences})
validation_df['Genotype'] = validation_df['Genotype'].apply(clean_label)
validation_df


fasta_path = "test.fasta"

genotypes, sequences = [], []

for record in SeqIO.parse(fasta_path, "fasta"):
    header = record.description.strip()
    seq = str(record.seq).replace("-", "")
    genotypes.append(header)
    sequences.append(seq)

# --- Clean the header into readable Genotype labels ---
def clean_label(header: str) -> str:

    # --- Extract species name ---
    species_match = re.search(r"(Trichophyton|Arthroderma)\s+[A-Za-z-]+", header)
    species = species_match.group(0) if species_match else "Unknown"

    # --- Extract Type number/roman numeral (e.g. Type_XIV, Type_II*, etc.) ---
    type_match = re.search(r"Type[_-]*([A-Za-z0-9*]+)", header, flags=re.IGNORECASE)
    if type_match:
        genotype_label = f"{species} {type_match.group(1).replace('_', '').replace('-', '')}"
    else:
        # fallback: no explicit Type_ — maybe a strain or isolate
        genotype_label = species

    return genotype_label.strip()

# --- Build dataframe ---
test_df = pd.DataFrame({
    "Genotype_Raw": genotypes,
    "Sequence": sequences
})
test_df["Genotype"] = test_df["Genotype_Raw"].apply(clean_label)
test_df['Sequence_length'] = test_df['Sequence'].str.len()
test_df
```

Out[125]:

|  | Genotype\_Raw | Sequence | Genotype | Sequence\_length |
| --- | --- | --- | --- | --- |
| 0 | T\_verrucosum LR890161.1 Trichophyton verrucosu... | ACGCGCAGGCCGGAGGCTGGCCCCCCACGATAGGGATCAGCGTTCC... | Trichophyton verrucosum | 590 |
| 1 | T\_africanum LR794140.1 Trichophyton benhamiae ... | ACGCGCAGGCCGGAGGCTGGCCCCCCACGATAGGCATCAAATGTTC... | Trichophyton benhamiae | 595 |
| 2 | T\_africanum LR794142.1 Trichophyton benhamiae ... | ACGCGCAGGCCGGAGGCTGGCCCCCCACGATAGGGATCAAATGTTC... | Trichophyton benhamiae | 595 |
| 3 | T\_africanum LR794141.1 Trichophyton benhamiae ... | ACGCGCAGGCCGGAGGCTGGCCCCCCACGATAGGGATCAAATGTTC... | Trichophyton benhamiae | 595 |
| 4 | T\_benhamiae LR794129.1 Trichophyton benhamiae ... | ACGCGCAGGCCGGAGGCTGGCCCCCCACGATAGGAATCAACGTTCC... | Trichophyton benhamiae | 591 |
| 5 | T\_bullosum LR794143.1 Trichophyton bullosum ge... | ACGCGCAGGCCGGAGGCTGGCCCCCCACGATAGGGATCAAATGTTC... | Trichophyton bullosum | 595 |
| 6 | T\_concentricum LR794126.1 Trichophyton concent... | ACGCGCAGGCCGGAGGCTGGCCCCCCACGATAGGAATCAACGTTCC... | Trichophyton concentricum | 591 |
| 7 | T\_erinacei LR794139.1 Trichophyton erinacei ge... | ACGCGCAGGCCGGAGGCTGGCCCCCCACGATAGGGATCAACGTTCC... | Trichophyton erinacei | 590 |
| 8 | T\_erinacei KJ606082.1 Trichophyton erinacei st... | ACGCGCAGGCCGGAGGCTGGCCCCCCACGATAGGGATCAACGTTCC... | Trichophyton erinacei | 591 |
| 9 | T\_eriotrephon FM992674.1 Trichophyton eriotrep... | ACGCGCAGGCCGGAGGCTGGCCCCCCACGATAGGGATCAACGTTCC... | Trichophyton eriotrephon | 594 |
| 10 | T\_europaeum LR794134.1 Trichophyton benhamiae ... | ACGCGCAGGCCGGAGGCTGGCCCCCCACGATAGGGACCAACGTTCC... | Trichophyton benhamiae | 592 |
| 11 | T\_europaeum LR794135.1 Trichophyton benhamiae ... | ACGCGCAGGCCGGAGGCTGGCCCCCCACGATAGGGATCAACGTTCC... | Trichophyton benhamiae | 592 |
| 12 | T\_japonicum LR794133.1 Trichophyton benhamiae ... | ACGCGCAGGCCGGAGGCTGGCCCCTCACGATAGGGACCAACGTTCC... | Trichophyton benhamiae | 592 |
| 13 | T\_japonicum LR794132.1 Trichophyton benhamiae ... | ACGCGCAGGCCGGAGGCTGGCCCCCCACGATAGGGACCAACGTTCC... | Trichophyton benhamiae | 592 |
| 14 | T\_persicum MW936609.1 Trichophyton sp. VH-2021... | ACGCGCAGGCCGGAGGCTGGCCCCCCACGATAGGGATCAACGTTCC... | Trichophyton sp | 592 |
| 15 | T\_spiraliforme MW936628.1 Trichophyton sp. VH-... | ACGCGCAGGCCGGAGGCTGGCCCCCCACGATAGGGATCAACGTTCC... | Trichophyton sp | 592 |
| 16 | T\_verrucosum LR890161.1 Trichophyton verrucosu... | ACGCGCAGGCCGGAGGCTGGCCCCCCACGATAGGGATCAGCGTTCC... | Trichophyton verrucosum | 590 |
| 17 | AB617775 Arthroderma vanbreuseghemii genes for... | ACGCGCAGGCCGGAGGCTGGCCCCCCACGATAGGGCCAAACGTCCG... | Arthroderma vanbreuseghemii | 595 |
| 18 | KU315316 Trichophyton interdigitale strain XQ3... | ACGCGCAGGCCGGAGGCTGGCCCCCCACGATAGGGCCAAACGTCCG... | Trichophyton interdigitale | 595 |
| 19 | MH859166 Trichophyton interdigitale strain CBS... | ACGCGCAGGCCGGAGGCTGGCCCCCCACGATAGGGCCAAACGTCCG... | Trichophyton interdigitale | 594 |
| 20 | FM986691\_Type\_I Trichophyton interdigitale ITS... | GCGCGCAGGCCGGAGGCTGGCCCCCCACGATAGGGCCAAACGTCCG... | Trichophyton interdigitale I | 596 |
| 21 | JX122216\_Type\_II Trichophyton interdigitale is... | GCGCGCAGGCCGGAGGCTGGCCCCCCACGATAGGGCCAAACGTCCG... | Trichophyton interdigitale II | 595 |
| 22 | KP132819\_Type\_II\*-XXIX Trichophyton interdigit... | ACGCGCAGGCCGGAGGCTGGCCCCCCACGATAGGGCCAAACGTCCG... | Trichophyton interdigitale II\* | 595 |
| 23 | FM986750\_Type\_III Trichophyton interdigitale I... | ACGCGCAGGCCGGAGGCTGGCCCCCCACGATAGGGCCAAACGTCCG... | Trichophyton interdigitale III | 595 |
| 24 | MF926358\_Type\_III\* Trichophyton mentagrophytes... | ACGCGCAGGCCGGAGGCTGGCCCCCCACGATAGGGCCAAACGTCCG... | Trichophyton mentagrophytes III\* | 595 |
| 25 | KJ606102\_Type\_IV Trichophyton mentagrophytes s... | ACGCGCAGGCCGGACGCTGGCCCCCCACGATAGGGCCAAACGTCCG... | Trichophyton mentagrophytes IV | 596 |
| 26 | KU496915\_Type\_V Trichophyton verrucosum strain... | ACGCGCAGGCCGGAGGCTGGCCCCCCACGATAGGGCCAAACGTCCG... | Trichophyton verrucosum V | 596 |
| 27 | KT285210\_Type\_VI Trichophyton mentagrophytes s... | ACGCGCAGGCCGGAGGCTGGCCCCCCACGATAGGGCCAAACGTCCG... | Trichophyton mentagrophytes VI | 595 |
| 28 | KT253558\_Type\_VII Trichophyton mentagrophytes ... | ACGCGCAGGCCGGAGGCTGGCCGCCCACGATAGGGCCAAACGTCCG... | Trichophyton mentagrophytes VII | 595 |
| 29 | KT192500\_Type\_VIII Trichophyton interdigitale ... | ACGCGCAGGCCGGAGGCTGGCCCCCCACGATAGGGCCAAACGTCCG... | Trichophyton interdigitale VIII | 595 |
| 30 | MK447613\_Type\_IX Trichophyton mentagrophytes s... | ACGCGCAGGCCGGAGGCTGGCCCCCCACGATAGGGCCAAACGTCCG... | Trichophyton mentagrophytes IX | 595 |
| 31 | MK312735\_Type\_X Trichophyton interdigitale str... | GCGCGCAGGCCGGAGGCTGGCCCCCCACGATAGGGCCAAACGTCCG... | Trichophyton interdigitale X | 595 |
| 32 | MK312755\_Type\_XI Trichophyton interdigitale st... | GCGCGCAGGCCGGAGGCTGGCCCCCCACGATAGGGCCAAACGTCCG... | Trichophyton interdigitale XI | 595 |
| 33 | MF109039\_Type\_XII-XXVII\_Salehi2021 Trichophyto... | GCGCGCAGGCCGGAGGCTGGCCCCCCACGATAGGGCCAAACGTCCG... | Trichophyton interdigitale XII | 595 |
| 34 | MK312917\_Type\_XIII Trichophyton mentagrophytes... | ACGCGCAGGCCGGAGGCTGGCCCCCCACGATAGGGCCAAACGTCCG... | Trichophyton mentagrophytes XIII | 595 |
| 35 | MK312950\_Type\_XIV Trichophyton mentagrophytes ... | ACGCGCAGGCCGGAGGCTGGCCCCCCACGATAGGGCCAAACGTCCG... | Trichophyton mentagrophytes XIV | 595 |
| 36 | MK312937\_Type\_XV Trichophyton mentagrophytes s... | ACGCGCAGGCCGGAGGCTGGCCCCCCACGATAGGGCCAAACGTCCG... | Trichophyton mentagrophytes XV | 595 |
| 37 | MK312933\_Type\_XVI Trichophyton mentagrophytes ... | ACGCGCAGGCCGGAGGCTGGCCCCCCACGATAGGGCCAAACGTCCG... | Trichophyton mentagrophytes XVI | 595 |
| 38 | MK312990\_Type\_XVII Trichophyton mentagrophytes... | ACGCGCAGGCCGGAGGCTGGCCCCCCACGATAGGGCCAAACGTCCG... | Trichophyton mentagrophytes XVII | 595 |
| 39 | MK313028\_Type\_XVIII-XXVIII\_Salehi2021 Trichoph... | ACGCGCAGGCCGGAGGCTGGCCCCCCACGATAGGGCCAAACGTCCG... | Trichophyton mentagrophytes XVIII | 595 |
| 40 | MK312878\_Type\_XIX Trichophyton mentagrophytes ... | ACGCGCAGGCCGGAGGCTGGCCCCCCACGATAGGGCCAAACGTCCG... | Trichophyton mentagrophytes XIX | 596 |
| 41 | MK313030\_Type\_XX Trichophyton mentagrophytes s... | ACGCGCAGGCCGGAGGCTGGCCCCCCACGATAGGGCCAAACGTCCG... | Trichophyton mentagrophytes XX | 597 |
| 42 | MK312891\_Type\_XXI Trichophyton mentagrophytes ... | ACGCGCAGGCCGGAGGCTGGCCCCCCACGATAGGGCCAAACGTCCG... | Trichophyton mentagrophytes XXI | 598 |
| 43 | MK312888\_Type\_XXII Trichophyton mentagrophytes... | ACGCGCAGGCCGGAGGCTGGCCCCCCACGATAGGGCCAAACGTCCG... | Trichophyton mentagrophytes XXII | 596 |
| 44 | MK313044\_Type\_XXIII Trichophyton mentagrophyte... | ACGCGCAGGCCGGAGGCTGGCCCCCCACGATAGGGCCAAACGTCCG... | Trichophyton mentagrophytes XXIII | 596 |
| 45 | AF170453\_Type\_XXIV Arthroderma vanbreuseghemii... | ACGCGCAGGCCGGAGGCTGGCCCCCCACGATAGGGCCAAACGTCCG... | Arthroderma vanbreuseghemii XXIV | 595 |
| 46 | MN886815\_Type\_XXV\_Nenoff2020 Trichophyton ment... | ACGCGCAGGCCGGAGGCTGGCCCCCCACGATAGGGCCAAACGTCCG... | Trichophyton mentagrophytes XXV | 595 |
| 47 | MT858874\_Type\_XXV\_Klinger2021 Trichophyton int... | GCGCGCAGGCCGGAGGCTGGCCCCCCACGATAGGGCCAAACGTCCG... | Trichophyton interdigitale XXV | 595 |
| 48 | KT253557\_Type\_XXVI\_Gnat2021 Trichophyton menta... | ACGCGCAGGCCGGAGGCTGGCCCCCCACGATAGGGCCAAACGTCCG... | Trichophyton mentagrophytes XXVI | 595 |
| 49 | MT858956\_Type\_XXVI\_Klinger2021 Trichophyton me... | ACGCGCAGGCCGGAGGCTGGCCCCCCACGATAGGGCCAAACGTCCG... | Trichophyton mentagrophytes XXVI | 595 |
| 50 | OM951158\_Type\_XXVII\_Uhrlass2022 Trichophyton m... | ACGCGCAGGCCGGAGGCTGGCCCCCCACGATAGGGCCAAACGTCCG... | Trichophyton mentagrophytes XXVII | 594 |
| 51 | OM951157\_Type\_XXVIII\_Uhrlass2022 Trichophyton ... | ACGCGCGGGCCGGACGCTGGCCCCCCACGATAGGGCCAAACGTCCG... | Trichophyton mentagrophytes XXVIII | 595 |

## Feature Engineering¶

In [126]:

```
import regex as re
def kmers_from_seq(seq, k=6):
    return " ".join([seq[i:i+k] for i in range(len(seq) - k + 1)])

def seqs_to_matrix(series):
    tokenised =series.apply(kmers_from_seq)
    vectorizer = CountVectorizer(tokenizer=str.split, ngram_range=(1,1), dtype=np.float32)
    X =vectorizer.fit_transform(tokenised).toarray()
    return X, vectorizer


#Input data
X_raw, vectorizer = seqs_to_matrix(df["Sequence"])
scaler = StandardScaler()
X = scaler.fit_transform(X_raw).astype("float32")[..., None]
X.shape
```

Out[126]:

```
(28, 683, 1)
```

In [127]:

```
label2id = {g: i for i, g in enumerate(sorted(df["Genotype"].unique()))}
y_int = df["Genotype"].map(label2id).to_numpy()
y = tf.keras.utils.to_categorical(y_int, num_classes=len(label2id))
y.shape
```

Out[127]:

```
(28, 28)
```

## Model Architecture¶

In [128]:

```
## Model Architecture


input_len = X.shape[1]

# Input layer
inputs = tf.keras.layers.Input(shape = (input_len, 1))

# First Convolution layer (32 filters)
x = tf.keras.layers.Conv1D(32, kernel_size=1, activation="relu")(inputs)

# Second Convolution layer (64  filter)
x = tf.keras.layers.Conv1D(64, kernel_size=1, activation="relu")(x)

# Flatten Layer
x = tf.keras.layers.Flatten()(x)

# Fully connected layer with relu
x = tf.keras.layers.Dense(500, activation="relu")(x)
x = tf.keras.layers.Dropout(0.10)(x)

#output layer
outputs = tf.keras.layers.Dense(y.shape[1], activation="softmax")(x)

# model
model = tf.keras.models.Model(inputs=inputs, outputs=outputs)

optimizer = tf.keras.optimizers.Adam(learning_rate=3e-4)
model.compile(optimizer=optimizer, loss="categorical_crossentropy", metrics=["accuracy"])

print(model.summary())
```

```
Model: "functional_8"
```

```
┏━━━━━━━━━━━━━━━━━━━━━━━━━━━━━━━━━┳━━━━━━━━━━━━━━━━━━━━━━━━┳━━━━━━━━━━━━━━━┓
┃ Layer (type)                    ┃ Output Shape           ┃       Param # ┃
┡━━━━━━━━━━━━━━━━━━━━━━━━━━━━━━━━━╇━━━━━━━━━━━━━━━━━━━━━━━━╇━━━━━━━━━━━━━━━┩
│ input_layer_4 (InputLayer)      │ (None, 683, 1)         │             0 │
├─────────────────────────────────┼────────────────────────┼───────────────┤
│ conv1d_8 (Conv1D)               │ (None, 683, 32)        │            64 │
├─────────────────────────────────┼────────────────────────┼───────────────┤
│ conv1d_9 (Conv1D)               │ (None, 683, 64)        │         2,112 │
├─────────────────────────────────┼────────────────────────┼───────────────┤
│ flatten_4 (Flatten)             │ (None, 43712)          │             0 │
├─────────────────────────────────┼────────────────────────┼───────────────┤
│ dense_8 (Dense)                 │ (None, 500)            │    21,856,500 │
├─────────────────────────────────┼────────────────────────┼───────────────┤
│ dropout_4 (Dropout)             │ (None, 500)            │             0 │
├─────────────────────────────────┼────────────────────────┼───────────────┤
│ dense_9 (Dense)                 │ (None, 28)             │        14,028 │
└─────────────────────────────────┴────────────────────────┴───────────────┘
```

```
 Total params: 21,872,704 (83.44 MB)
```

```
 Trainable params: 21,872,704 (83.44 MB)
```

```
 Non-trainable params: 0 (0.00 B)
```

```
None
```

In [129]:

```
# Train the cnn model
early_stop = tf.keras.callbacks.EarlyStopping(
    monitor="val_loss",
    patience=10,
    restore_best_weights=True
)

history = model.fit(
    X, y,
    epochs=100,
    batch_size=10,
    verbose=2

)
```

```
Epoch 1/100
3/3 - 1s - 308ms/step - accuracy: 0.0000e+00 - loss: 3.5406
Epoch 2/100
3/3 - 0s - 68ms/step - accuracy: 0.5000 - loss: 2.6228
Epoch 3/100
3/3 - 0s - 64ms/step - accuracy: 0.6429 - loss: 1.9968
Epoch 4/100
3/3 - 0s - 62ms/step - accuracy: 0.6786 - loss: 1.5659
Epoch 5/100
3/3 - 0s - 62ms/step - accuracy: 0.8214 - loss: 1.2449
Epoch 6/100
3/3 - 0s - 63ms/step - accuracy: 0.8929 - loss: 1.0355
Epoch 7/100
3/3 - 0s - 66ms/step - accuracy: 0.8214 - loss: 0.9061
Epoch 8/100
3/3 - 0s - 67ms/step - accuracy: 0.8571 - loss: 0.7662
Epoch 9/100
3/3 - 0s - 66ms/step - accuracy: 0.9286 - loss: 0.6061
Epoch 10/100
3/3 - 0s - 65ms/step - accuracy: 0.9286 - loss: 0.5334
Epoch 11/100
3/3 - 0s - 69ms/step - accuracy: 0.9286 - loss: 0.4785
Epoch 12/100
3/3 - 0s - 66ms/step - accuracy: 0.9643 - loss: 0.3764
Epoch 13/100
3/3 - 0s - 69ms/step - accuracy: 1.0000 - loss: 0.3358
Epoch 14/100
3/3 - 0s - 60ms/step - accuracy: 1.0000 - loss: 0.2997
Epoch 15/100
3/3 - 0s - 62ms/step - accuracy: 0.9643 - loss: 0.2806
Epoch 16/100
3/3 - 0s - 66ms/step - accuracy: 0.8929 - loss: 0.2904
Epoch 17/100
3/3 - 0s - 65ms/step - accuracy: 0.9643 - loss: 0.2326
Epoch 18/100
3/3 - 0s - 66ms/step - accuracy: 1.0000 - loss: 0.2159
Epoch 19/100
3/3 - 0s - 62ms/step - accuracy: 0.9286 - loss: 0.2115
Epoch 20/100
3/3 - 0s - 67ms/step - accuracy: 0.9286 - loss: 0.1525
Epoch 21/100
3/3 - 0s - 67ms/step - accuracy: 0.9643 - loss: 0.1626
Epoch 22/100
3/3 - 0s - 68ms/step - accuracy: 1.0000 - loss: 0.1303
Epoch 23/100
3/3 - 0s - 65ms/step - accuracy: 1.0000 - loss: 0.1137
Epoch 24/100
3/3 - 0s - 65ms/step - accuracy: 1.0000 - loss: 0.1000
Epoch 25/100
3/3 - 0s - 66ms/step - accuracy: 1.0000 - loss: 0.0864
Epoch 26/100
3/3 - 0s - 59ms/step - accuracy: 0.9643 - loss: 0.0960
Epoch 27/100
3/3 - 0s - 64ms/step - accuracy: 1.0000 - loss: 0.0776
Epoch 28/100
3/3 - 0s - 66ms/step - accuracy: 1.0000 - loss: 0.0803
Epoch 29/100
3/3 - 0s - 61ms/step - accuracy: 0.9643 - loss: 0.0768
Epoch 30/100
3/3 - 0s - 61ms/step - accuracy: 0.9643 - loss: 0.1189
Epoch 31/100
3/3 - 0s - 60ms/step - accuracy: 1.0000 - loss: 0.0625
Epoch 32/100
3/3 - 0s - 61ms/step - accuracy: 1.0000 - loss: 0.0565
Epoch 33/100
3/3 - 0s - 71ms/step - accuracy: 1.0000 - loss: 0.0444
Epoch 34/100
3/3 - 0s - 66ms/step - accuracy: 1.0000 - loss: 0.0672
Epoch 35/100
3/3 - 0s - 65ms/step - accuracy: 0.9643 - loss: 0.0740
Epoch 36/100
3/3 - 0s - 62ms/step - accuracy: 1.0000 - loss: 0.0440
Epoch 37/100
3/3 - 0s - 63ms/step - accuracy: 1.0000 - loss: 0.0456
Epoch 38/100
3/3 - 0s - 63ms/step - accuracy: 1.0000 - loss: 0.0385
Epoch 39/100
3/3 - 0s - 67ms/step - accuracy: 1.0000 - loss: 0.0521
Epoch 40/100
3/3 - 0s - 62ms/step - accuracy: 1.0000 - loss: 0.0279
Epoch 41/100
3/3 - 0s - 72ms/step - accuracy: 1.0000 - loss: 0.0300
Epoch 42/100
3/3 - 0s - 69ms/step - accuracy: 1.0000 - loss: 0.0408
Epoch 43/100
3/3 - 0s - 67ms/step - accuracy: 1.0000 - loss: 0.0263
Epoch 44/100
3/3 - 0s - 65ms/step - accuracy: 1.0000 - loss: 0.0256
Epoch 45/100
3/3 - 0s - 60ms/step - accuracy: 1.0000 - loss: 0.0288
Epoch 46/100
3/3 - 0s - 66ms/step - accuracy: 1.0000 - loss: 0.0300
Epoch 47/100
3/3 - 0s - 64ms/step - accuracy: 1.0000 - loss: 0.0310
Epoch 48/100
3/3 - 0s - 65ms/step - accuracy: 1.0000 - loss: 0.0255
Epoch 49/100
3/3 - 0s - 62ms/step - accuracy: 1.0000 - loss: 0.0268
Epoch 50/100
3/3 - 0s - 61ms/step - accuracy: 1.0000 - loss: 0.0183
Epoch 51/100
3/3 - 0s - 63ms/step - accuracy: 1.0000 - loss: 0.0218
Epoch 52/100
3/3 - 0s - 63ms/step - accuracy: 1.0000 - loss: 0.0187
Epoch 53/100
3/3 - 0s - 61ms/step - accuracy: 1.0000 - loss: 0.0230
Epoch 54/100
3/3 - 0s - 65ms/step - accuracy: 1.0000 - loss: 0.0266
Epoch 55/100
3/3 - 0s - 62ms/step - accuracy: 1.0000 - loss: 0.0135
Epoch 56/100
3/3 - 0s - 62ms/step - accuracy: 1.0000 - loss: 0.0151
Epoch 57/100
3/3 - 0s - 63ms/step - accuracy: 1.0000 - loss: 0.0160
Epoch 58/100
3/3 - 0s - 62ms/step - accuracy: 1.0000 - loss: 0.0159
Epoch 59/100
3/3 - 0s - 62ms/step - accuracy: 1.0000 - loss: 0.0108
Epoch 60/100
3/3 - 0s - 65ms/step - accuracy: 1.0000 - loss: 0.0141
Epoch 61/100
3/3 - 0s - 61ms/step - accuracy: 1.0000 - loss: 0.0147
Epoch 62/100
3/3 - 0s - 61ms/step - accuracy: 1.0000 - loss: 0.0173
Epoch 63/100
3/3 - 0s - 62ms/step - accuracy: 1.0000 - loss: 0.0089
Epoch 64/100
3/3 - 0s - 63ms/step - accuracy: 1.0000 - loss: 0.0087
Epoch 65/100
3/3 - 0s - 61ms/step - accuracy: 1.0000 - loss: 0.0102
Epoch 66/100
3/3 - 0s - 61ms/step - accuracy: 1.0000 - loss: 0.0107
Epoch 67/100
3/3 - 0s - 62ms/step - accuracy: 1.0000 - loss: 0.0221
Epoch 68/100
3/3 - 0s - 66ms/step - accuracy: 1.0000 - loss: 0.0082
Epoch 69/100
3/3 - 0s - 65ms/step - accuracy: 1.0000 - loss: 0.0095
Epoch 70/100
3/3 - 0s - 63ms/step - accuracy: 1.0000 - loss: 0.0076
Epoch 71/100
3/3 - 0s - 63ms/step - accuracy: 1.0000 - loss: 0.0044
Epoch 72/100
3/3 - 0s - 60ms/step - accuracy: 1.0000 - loss: 0.0090
Epoch 73/100
3/3 - 0s - 62ms/step - accuracy: 1.0000 - loss: 0.0082
Epoch 74/100
3/3 - 0s - 62ms/step - accuracy: 1.0000 - loss: 0.0079
Epoch 75/100
3/3 - 0s - 67ms/step - accuracy: 1.0000 - loss: 0.0077
Epoch 76/100
3/3 - 0s - 62ms/step - accuracy: 1.0000 - loss: 0.0093
Epoch 77/100
3/3 - 0s - 63ms/step - accuracy: 1.0000 - loss: 0.0087
Epoch 78/100
3/3 - 0s - 62ms/step - accuracy: 1.0000 - loss: 0.0080
Epoch 79/100
3/3 - 0s - 61ms/step - accuracy: 1.0000 - loss: 0.0060
Epoch 80/100
3/3 - 0s - 59ms/step - accuracy: 1.0000 - loss: 0.0067
Epoch 81/100
3/3 - 0s - 64ms/step - accuracy: 1.0000 - loss: 0.0107
Epoch 82/100
3/3 - 0s - 61ms/step - accuracy: 1.0000 - loss: 0.0146
Epoch 83/100
3/3 - 0s - 61ms/step - accuracy: 1.0000 - loss: 0.0041
Epoch 84/100
3/3 - 0s - 64ms/step - accuracy: 1.0000 - loss: 0.0114
Epoch 85/100
3/3 - 0s - 66ms/step - accuracy: 1.0000 - loss: 0.0069
Epoch 86/100
3/3 - 0s - 64ms/step - accuracy: 1.0000 - loss: 0.0072
Epoch 87/100
3/3 - 0s - 63ms/step - accuracy: 1.0000 - loss: 0.0080
Epoch 88/100
3/3 - 0s - 60ms/step - accuracy: 1.0000 - loss: 0.0083
Epoch 89/100
3/3 - 0s - 63ms/step - accuracy: 1.0000 - loss: 0.0056
Epoch 90/100
3/3 - 0s - 65ms/step - accuracy: 1.0000 - loss: 0.0070
Epoch 91/100
3/3 - 0s - 61ms/step - accuracy: 1.0000 - loss: 0.0046
Epoch 92/100
3/3 - 0s - 68ms/step - accuracy: 1.0000 - loss: 0.0064
Epoch 93/100
3/3 - 0s - 64ms/step - accuracy: 1.0000 - loss: 0.0050
Epoch 94/100
3/3 - 0s - 65ms/step - accuracy: 1.0000 - loss: 0.0043
Epoch 95/100
3/3 - 0s - 64ms/step - accuracy: 1.0000 - loss: 0.0031
Epoch 96/100
3/3 - 0s - 65ms/step - accuracy: 1.0000 - loss: 0.0066
Epoch 97/100
3/3 - 0s - 64ms/step - accuracy: 1.0000 - loss: 0.0040
Epoch 98/100
3/3 - 0s - 64ms/step - accuracy: 1.0000 - loss: 0.0049
Epoch 99/100
3/3 - 0s - 63ms/step - accuracy: 1.0000 - loss: 0.0034
Epoch 100/100
3/3 - 0s - 66ms/step - accuracy: 1.0000 - loss: 0.0039
```

## Model Accuracy and Model Loss¶

In [130]:

```
import matplotlib.pyplot as plt

def plot_training_history(history_obj):
    history_dict = history_obj.history
    acc = history_dict.get("accuracy", [])
    val_acc = history_dict.get("val_accuracy", [])
    loss = history_dict.get("loss", [])
    val_loss = history_dict.get("val_loss", [])
    epochs_range = range(1, len(acc) + 1)
    plt.figure(figsize=(10, 4), dpi=150)

    # Accuracy plot
    plt.subplot(1, 2, 1)
    plt.plot(epochs_range, acc, label="Training Accuracy")
    if val_acc:
        plt.plot(epochs_range, val_acc, label="Validation Accuracy")
    plt.title("Model Accuracy")
    plt.xlabel("Epoch")
    plt.ylabel("Accuracy")
    plt.grid(True)
    plt.legend()

    # Loss plot
    plt.subplot(1, 2, 2)
    plt.plot(epochs_range, loss, label="Training Loss")
    if val_loss:
        plt.plot(epochs_range, val_loss, label="Validation Loss")
    plt.title("Model Loss")
    plt.xlabel("Epoch")
    plt.ylabel("Loss")
    plt.grid(True)
    plt.legend()

    plt.tight_layout()
    plt.show()

plot_training_history(history)
```

## Out of Distribution detection¶

In [131]:

```
from sklearn.covariance import LedoitWolf
import numpy as np

# Extracting Embeddings from dense layer
embed_model = tf.keras.Model(inputs=model.input, outputs=model.layers[-2].output)

#getting embeddings for training samples
F_train = embed_model.predict(X, verbose=0)  # This will be no of classes * dense layer size i.e., 28 * 500
y_train_labels = np.argmax(y, axis=1)  # will return class labels in one dimension array
num_classes = y.shape[1]  #28 classes


# Computing mean of the embeddings
means = np.stack([F_train[y_train_labels == c].mean(axis=0) for c in range(num_classes)])
means
```

Out[131]:

```
array([[0.0000000e+00, 0.0000000e+00, 9.2668418e-04, ..., 7.2295731e-01,
        1.3033447e-01, 3.2445920e+00],
       [0.0000000e+00, 0.0000000e+00, 0.0000000e+00, ..., 1.7959273e+00,
        0.0000000e+00, 6.5527242e-01],
       [0.0000000e+00, 0.0000000e+00, 0.0000000e+00, ..., 2.2297325e+00,
        0.0000000e+00, 1.3776788e+00],
       ...,
       [0.0000000e+00, 0.0000000e+00, 0.0000000e+00, ..., 3.0230558e+00,
        1.5506617e+00, 1.5787783e+00],
       [0.0000000e+00, 0.0000000e+00, 0.0000000e+00, ..., 5.0282536e+00,
        0.0000000e+00, 9.8878384e-01],
       [0.0000000e+00, 0.0000000e+00, 0.0000000e+00, ..., 3.6023402e+00,
        5.8888775e-01, 6.6586095e-01]], dtype=float32)
```

# Ledoit-Wolf Covariance Estimator¶

The LedoitWolf method from sklearn.covariance calculates a **more stable covariance matrix** using **shrinkage**
Applied when

- You have many features but few samples
- The standard covariance (`np.cov`) is noisy or unstable.

In [132]:

```
cov = LedoitWolf().fit(F_train).covariance_ 
prec = np.linalg.pinv(cov) #It returns the pseudo-inverse of the input matrix 
prec
```

Out[132]:

```
array([[ 2.0042155e+00,  0.0000000e+00,  0.0000000e+00, ...,
         0.0000000e+00,  0.0000000e+00,  0.0000000e+00],
       [ 0.0000000e+00,  2.0042155e+00,  0.0000000e+00, ...,
         0.0000000e+00,  0.0000000e+00,  0.0000000e+00],
       [ 0.0000000e+00,  0.0000000e+00,  1.9859362e+00, ...,
         6.1884373e-03, -4.2029247e-03,  7.9721212e-05],
       ...,
       [ 0.0000000e+00,  0.0000000e+00,  6.1884448e-03, ...,
         1.8591144e+00,  1.5898332e-02,  3.1038869e-02],
       [ 0.0000000e+00,  0.0000000e+00, -4.2029321e-03, ...,
         1.5898339e-02,  1.8997819e+00,  1.7695429e-02],
       [ 0.0000000e+00,  0.0000000e+00,  7.9702586e-05, ...,
         3.1038865e-02,  1.7695405e-02,  1.8574706e+00]], dtype=float32)
```

# Prediction Pipeline¶

In [133]:

```
tau_maha = 0.0007 

tokens = [kmers_from_seq(seq) for seq in validation_df["Sequence"]]
X_mat = vectorizer.transform(tokens).toarray()
X_mat = (X_mat - scaler.mean_) / scaler.scale_
features = X_mat.astype("float32")[..., None]
probs = model.predict(features, batch_size=20, verbose=0)
pred_ids = np.argmax(probs, axis=1)
confidences = np.max(probs, axis=1)
id2label = {i: g for g, i in label2id.items()}
pred_labels = [id2label[i] for i in pred_ids]


F_valid = embed_model.predict(features, verbose=0)


maha_scores = []
for f in F_valid:
    diffs = means - f[None, :] 
    d2 = np.einsum("nd,dd,nd->n", diffs, prec, diffs)
    maha_scores.append(np.sqrt(d2.min()))
maha_scores = np.array(maha_scores)


validity = np.where(maha_scores <= tau_maha,
                    "In class Distribution",
                    "Out of Distribution")

# Suppress predicted labels for OOD
final_preds = [lbl if v.startswith("In") else "N/A"
               for lbl, v in zip(pred_labels, validity)]


validation_df["Predicted_Genotype"] = final_preds
validation_df["Confidence"] = np.round(confidences, 6)
validation_df["Mahalanobis"] = np.round(maha_scores, 6)
validation_df["Validity"] = validity
validation_df
```

Out[133]:

|  | Genotype | Sequence | Predicted\_Genotype | Confidence | Mahalanobis | Validity |
| --- | --- | --- | --- | --- | --- | --- |
| 0 | Trichophyton interdigitale I | GCGCGCAGGCCGGAGGCTGGCCCCCCACGATAGGGCCAAACGTCCG... | Trichophyton interdigitale I | 0.995316 | 0.000003 | In class Distribution |
| 1 | Trichophyton interdigitale II | GCGCGCAGGCCGGAGGCTGGCCCCCCACGATAGGGCCAAACGTCCG... | Trichophyton interdigitale II | 0.965935 | 0.000002 | In class Distribution |
| 2 | Trichophyton mentagrophytes II\* | ACGCGCAGGCCGGAGGCTGGCCCCCCACGATAGGGCCAAACGTCCG... | Trichophyton mentagrophytes II\* | 0.988352 | 0.000002 | In class Distribution |
| 3 | Trichophyton mentagrophytes III | ACGCGCAGGCCGGAGGCTGGCCCCCCACGATAGGGCCAAACGTCCG... | Trichophyton mentagrophytes III | 1.000000 | 0.000005 | In class Distribution |
| 4 | Trichophyton mentagrophytes III\* | ACGCGCAGGCCGGAGGCTGGCCCCCCACGATAGGGCCAAACGTCCG... | Trichophyton mentagrophytes III\* | 0.995671 | 0.000002 | In class Distribution |
| 5 | Trichophyton mentagrophytes IV | ACGCGCAGGCCGGACGCTGGCCCCCCACGATAGGGCCAAACGTCCG... | Trichophyton mentagrophytes IV | 0.999982 | 0.000004 | In class Distribution |
| 6 | Trichophyton mentagrophytes V | ACGCGCAGGCCGGAGGCTGGCCCCCCACGATAGGGCCAAACGTCCG... | Trichophyton mentagrophytes V | 0.992988 | 0.000002 | In class Distribution |
| 7 | Trichophyton mentagrophytes VI | ACGCGCAGGCCGGAGGCTGGCCCCCCACGATAGGGCCAAACGTCCG... | Trichophyton mentagrophytes VI | 0.999966 | 0.000004 | In class Distribution |
| 8 | Trichophyton mentagrophytes VII | ACGCGCAGGCCGGAGGCTGGCCGCCCACGATAGGGCCAAACGTCCG... | Trichophyton mentagrophytes VII | 1.000000 | 0.000006 | In class Distribution |
| 9 | Trichophyton indotineae | ACGCGCAGGCCGGAGGCTGGCCCCCCACGATAGGGCCAAACGTCCG... | Trichophyton indotineae | 0.999917 | 0.000001 | In class Distribution |
| 10 | Trichophyton mentagrophytes IX | ACGCGCAGGCCGGAGGCTGGCCCCCCACGATAGGGCCAAACGTCCG... | Trichophyton mentagrophytes IX | 0.999998 | 0.000005 | In class Distribution |
| 11 | Trichophyton interdigitale X | GCGCGCAGGCCGGAGGCTGGCCCCCCACGATAGGGCCAAACGTCCG... | Trichophyton interdigitale X | 0.999971 | 0.000005 | In class Distribution |
| 12 | Trichophyton interdigitale XI | GCGCGCAGGCCGGAGGCTGGCCCCCCACGATAGGGCCAAACGTCCG... | Trichophyton interdigitale XI | 0.997620 | 0.000001 | In class Distribution |
| 13 | Trichophyton interdigitale XII | GCGCGCAGGCCGGAGGCTGGCCCCCCACGATAGGGCCAAACGTCCG... | Trichophyton interdigitale XII | 0.995967 | 0.000001 | In class Distribution |
| 14 | Trichophyton mentagrophytes XIII | ACGCGCAGGCCGGAGGCTGGCCCCCCACGATAGGGCCAAACGTCCG... | Trichophyton mentagrophytes XIII | 1.000000 | 0.000007 | In class Distribution |
| 15 | Trichophyton mentagrophytes XIV | ACGCGCAGGCCGGAGGCTGGCCCCCCACGATAGGGCCAAACGTCCG... | Trichophyton mentagrophytes XIV | 0.999992 | 0.000004 | In class Distribution |
| 16 | Trichophyton mentagrophytes XV | ACGCGCAGGCCGGAGGCTGGCCCCCCACGATAGGGCCAAACGTCCG... | Trichophyton mentagrophytes XV | 0.999970 | 0.000004 | In class Distribution |
| 17 | Trichophyton mentagrophytes XVI | ACGCGCAGGCCGGAGGCTGGCCCCCCACGATAGGGCCAAACGTCCG... | Trichophyton mentagrophytes XVI | 0.999717 | 0.000002 | In class Distribution |
| 18 | Trichophyton mentagrophytes XVII | ACGCGCAGGCCGGAGGCTGGCCCCCCACGATAGGGCCAAACGTCCG... | Trichophyton mentagrophytes XVII | 0.999980 | 0.000004 | In class Distribution |
| 19 | Trichophyton mentagrophytes XVIII | ACGCGCAGGCCGGAGGCTGGCCCCCCACGATAGGGCCAAACGTCCG... | Trichophyton mentagrophytes XVIII | 0.994246 | 0.000001 | In class Distribution |
| 20 | Trichophyton mentagrophytes XIX | ACGCGCAGGCCGGAGGCTGGCCCCCCACGATAGGGCCAAACGTCCG... | Trichophyton mentagrophytes XIX | 0.995479 | 0.000001 | In class Distribution |
| 21 | Trichophyton mentagrophytes XX | ACGCGCAGGCCGGAGGCTGGCCCCCCACGATAGGGCCAAACGTCCG... | Trichophyton mentagrophytes XX | 0.983299 | 0.000002 | In class Distribution |
| 22 | Trichophyton mentagrophytes XXI | ACGCGCAGGCCGGAGGCTGGCCCCCCACGATAGGGCCAAACGTCCG... | Trichophyton mentagrophytes XXI | 0.998826 | 0.000003 | In class Distribution |
| 23 | Trichophyton mentagrophytes XXII | ACGCGCAGGCCGGAGGCTGGCCCCCCACGATAGGGCCAAACGTCCG... | Trichophyton mentagrophytes XXII | 0.999961 | 0.000004 | In class Distribution |
| 24 | Trichophyton mentagrophytes XXIII | ACGCGCAGGCCGGAGGCTGGCCCCCCACGATAGGGCCAAACGTCCG... | Trichophyton mentagrophytes XXIII | 0.999973 | 0.000004 | In class Distribution |
| 25 | Trichophyton mentagrophytes XXIV | ACGCGCAGGCCGGAGGCTGGCCCCCCACGATAGGGCCAAACGTCCG... | Trichophyton mentagrophytes XXIV | 0.995898 | 0.000002 | In class Distribution |
| 26 | interdigitale XXV | GCGCGCAGGCCGGAGGCTGGCCCCCCACGATAGGGCCAAACGTCCG... | interdigitale XXV | 0.999988 | 0.000005 | In class Distribution |
| 27 | Trichophyton mentagrophytes XXVI | ACGCGCAGGCCGGAGGCTGGCCCCCCACGATAGGGCCAAACGTCCG... | Trichophyton mentagrophytes XXVI | 1.000000 | 0.000006 | In class Distribution |

## Raw .ab1 reads from Sanger sequencing frequently include extra bases apart from the ITS region,so, trimming was performed using conserved start and end motifs to retain only the canonical ITS sequence.¶

In [134]:

```
import regex
def trim_seq(seq):
    seq = str(seq).upper()
    start_pattern = r'(?:[GA]CGCGCAGGCCGGA[GC]GCTGGCC[GC]CCCACGA)'
    end_pattern   = r'(?:GGCCTCA[AG]AATCTGTTTTATACTTAT[TC][GA])'
    sm = regex.search(start_pattern, seq)
    em = regex.search(end_pattern, seq)
    if sm and em and sm.start() < em.end():
        return seq[sm.start():em.end()]
    if sm and not em:
        return seq[sm.start():]
    if em and not sm:
        return seq[:em.end()]
    return seq
```

In [141]:

```
import numpy as np
import tensorflow as tf

tau_maha =1

sequence = """TTTTTTTACGCGCAGGCCGGAGGCTGGCCCCCCACGATAGGGCCAAACGTCCGTCAGGGGTGAGCAGATGTGCGCCGGCCGTACCGCCCCATTCTTGTCTACCTTACTCGGTTGCCTCGGCGGGCCGCGCTCTCTCAGGAGAGCCGTTCGGCGAGCCTCTCTTTAGTGGCTCAACGCTGGACCGCGCCCGCCGGAGGACAGACGCAAAAAAATTCTTTCAGAAGAGCTGTCAGTCTGAGCGTTAGCAAGCAAAAATCAGTTAAAACTTTCAACAACGGATCTCTTGGTTCCGGCATCGATGAAGAACGCAGCGAAATGCGATAAGTAATGTGAATTGCAGAATTCCGTGAATCATCGAATCTTTGAACGCACATTGCGCCCCCTGGCATTCCGGGGGGCATGCCTGTTCGAGCGTCATTTCAGCCCCCTCAAGCCCGGCTTGTGTGATGGACGATCGTCCGGCGCCCCCGTCTTTGGGGGTGCGGGACGCGCCCGAAAAGCAGTGGCCAGGCCGCGATTCCGGCTTCCTAGGCGAATGGGCAACAAACCAGCGCCTCCAGGACCGGCCGCCCTGGCCTCAGAATCTGTTTTATACTTATCATTTTT""".replace('\n', '')

sequences = trim_seq(sequence)

print(len(sequences))

token = kmers_from_seq(sequence)
X = vectorizer.transform([token]).toarray()
X = (X - scaler.mean_) / scaler.scale_
X_patient = X.astype("float32")[..., None]


probs = model.predict(X_patient, verbose=0)
pred_id = int(np.argmax(probs[0]))
confidence = float(np.max(probs[0]))
pred_label = id2label[pred_id]


F_patient = embed_model.predict(X_patient, verbose=0)[0]
diffs = means - F_patient[None, :]
d2 = np.einsum("nd,dd,nd->n", diffs, prec, diffs)
mahalanobis = float(np.sqrt(d2.min()))


if mahalanobis <= tau_maha:
    validity = "In-Distribution"
    final_pred = pred_label
else:
    validity = "Out-of-Distribution"
    final_pred = "N/A"


print(f"{validity}")
print(f"Confidence   : {confidence:.4f}")
print(f"Mahalanobis Dist.  : {mahalanobis:.6f}")
print(f"Predicted Genotype : {final_pred}")
```

```
594
In-Distribution
Confidence   : 1.0000
Mahalanobis Dist.  : 0.295356
Predicted Genotype : Trichophyton mentagrophytes XXIII
```

## Out-ofistribution (OOD) example¶

In [143]:

```
import numpy as np
import tensorflow as tf

tau_maha =1

sequence = """ACGCGCAGGCCGGAGGCTGGCCCCCCACGATAGGGACCGACGTTCCATCAGGGGTGAGCAGACGTGCGCCGGCCGTACGCCCCCATTCTTGTCTACCTCACCCGGTTGCCTCGGCGGGCCGCGCTCCCCCTGCCAGGGAGAGCCGTCCGGCGGGCCCCTTCTGGGAGCCTCGAGCCGGACCGCGCCCGCCGGAGGACAGACACCAAGAAAAAATTCTCTGAAGAGCTGTCAGTCTGAGCGTTTAGCAAGCACAATCAGTTAAAACTTTCAACAACGGATCTCTTGGTTCCGGCATCGATGAAGAACGCAGCGAAATGCGATAAGTAATGTGAATTGCAGAATTCCGTGAATCATCGAATCTTTGAACGCACATTGCGCCCTCTGGCATTCCGGGGGGCATGCCTGTTCGAGCGTCATTTCAACCCCTCAAGCCCGGCTTGTGTGATGGACGACCGTCCGGCCCCTCCCTTCGGGGGCGGGACGCGCCCGAAAAGCAGTGGCCAGGCCGCGATTCCGGCTTCCTAGGCGAATGGGCAGCCAATTCAGCGCCCTCAGGACCGGCCGCCCTGGCCCCAATCTTTATATATATATATATCTTTTCAGGTTGACCTCGGATCAGG""".replace('\n', '')

sequences = trim_seq(sequence)

print(len(sequences))

token = kmers_from_seq(sequence)
X = vectorizer.transform([token]).toarray()
X = (X - scaler.mean_) / scaler.scale_
X_patient = X.astype("float32")[..., None]


probs = model.predict(X_patient, verbose=0)
pred_id = int(np.argmax(probs[0]))
confidence = float(np.max(probs[0]))
pred_label = id2label[pred_id]


F_patient = embed_model.predict(X_patient, verbose=0)[0]
diffs = means - F_patient[None, :]
d2 = np.einsum("nd,dd,nd->n", diffs, prec, diffs)
mahalanobis = float(np.sqrt(d2.min()))


if mahalanobis <= tau_maha:
    validity = "In-Distribution"
    final_pred = pred_label
else:
    validity = "Out-of-Distribution"
    final_pred = "N/A"


print(f"{validity}")
print(f"Confidence   : {confidence:.4f}")
print(f"Mahalanobis Dist.  : {mahalanobis:.6f}")
print(f"Predicted Genotype : {final_pred}")
```

```
620
Out-of-Distribution
Confidence   : 0.5009
Mahalanobis Dist.  : 57.671654
Predicted Genotype : N/A
```

In [137]:

```
patient_seq
```

Out[137]:

|  | name | sequence | Query\_Sequence\_length |
| --- | --- | --- | --- |
| 0 | patientAA | AACAAGGTTTCCGTAGGTGAACCTGCGGAAGGATCATTAACGCGCA... | 646 |
| 1 | patientAB | ACAAGGTTTCCGTAGGTGAACCTGCGGAAGGATCATTAACGCGCAG... | 644 |
| 2 | patientAC | AACAAGGTTTCCGTAGGTGAACCTGCGGAAGGATCATTAACGCGCA... | 641 |
| 3 | patientAD | AACAAGGTTTCCGTAGGTGAACCTGCGGAAGGATCATTAACGCGCA... | 641 |
| 4 | patientAE | AACAAGGTTTCCGTAGGTGAACCTGCGGAAGGATCATTAACGCGCA... | 649 |
| 5 | patientAF | AACAAGGTTTCCGTAGGTGAACCTGCGGAAGGATCATTAACGCGCA... | 641 |
| 6 | patientAG | GTCGTAACAAGGTTTCCGTAGGTGAACCTGCGGAAGGATCATTAAC... | 650 |
| 7 | patientAH | ACAAGGTTTCCGTAGGTGAACCTGCGGAAGGATCATTAACGCGCAG... | 642 |
| 8 | patientAI | TAACAAGGTTTCCGTAGGTGAACCTGCGGAAGGATCATTAACGCGC... | 641 |
| 9 | patientAJ | TAACAAGGTTTCCGTAGGTGAACCTGCGGAAGGATCATTAACGCGC... | 642 |
| 10 | patientAK | TAACAAGGTTTCCGTAGGTGAACCTGCGGAAGGATCATTAACGCGC... | 643 |
| 11 | patientAL | TAACAAGGTTTCCGTAGGTGAACCTGCGGAAGGATCATTAACGCGC... | 642 |
| 12 | patientAM | TAACAAGGTTTCCGTAGGTGAACCTGCGGAAGGATCATTAACGCGC... | 643 |
| 13 | patientAN | AGTAAAAAGTCGTAACAAGGTTTCCGTAGGTGAACCTGCGGAAGGA... | 656 |
| 14 | patientAO | AACAAGGTTTCCGTAGGTGAACCTGCGGAAGGATCATTAACGCGCA... | 641 |
| 15 | patientAP | GTCGTAACAAGGTTTCCGTAGGTGAACCTGCGGAAGGATCATTAAC... | 654 |
| 16 | patientAQ | GTAACAAGGTTTCCGTAGGTGAACCTGCGGAAGGATCATTAGCGCG... | 651 |
| 17 | patientAR | GTAACAAGGTTTCCGTAGGTGAACCTGCGGAAGGATCATTAGCGCG... | 645 |
| 18 | patientAS | AACAAGGTTTCCGTAGGTGAACCTGCGGAAGGATCATTAACGCGCA... | 648 |
| 19 | patientAT | TAACAAGGTTTCCGTAGGTGAACCTGCGGAAGGATCATTAACGCGC... | 644 |
| 20 | patientAU | TCGTAACAAGGTTTCCGTAGGTGAACCTGCGGAAGGATCATTAACG... | 647 |
| 21 | patientA | GCGGAAGGATCATTAGCGCGCAGGCCGGAGGCTGGCCCCCCACGAT... | 619 |
| 22 | PatientBA | TGATTCAAAACAAAAACAAGTCAAAACTTTTAACAACGGATCTCTT... | 362 |
| 23 | PatientBB | TAACAAGGTTTCCGTAGGTGAACCTGCGGAAGGATCATTAACGCGC... | 660 |
| 24 | patientBC | TAACAAGGTTTCCGTAGGTGAACCTGCGGAAGGATCATTACCGAGT... | 548 |

In [144]:

```
# applying trimming the ends of the sequence
patient_seq['sequence'] = patient_seq['sequence'].apply(trim_seq)
patient_seq['Query_Sequence_length'] = patient_seq['sequence'].str.len()
tokens = [kmers_from_seq(seq) for seq in patient_seq["sequence"]]
X_mat = vectorizer.transform(tokens).toarray()
X_mat = (X_mat - scaler.mean_) / scaler.scale_
features = X_mat.astype("float32")[..., None]


probs = model.predict(features, batch_size=20, verbose=0)
pred_ids = np.argmax(probs, axis=1)
confidences = np.max(probs, axis=1)
pred_labels = [id2label[i] for i in pred_ids]


F_test = embed_model.predict(features, verbose=0)


maha_scores = []
for f in F_test:
    diffs = means - f[None, :]
    d2 = np.einsum("nd,dd,nd->n", diffs, prec, diffs)
    maha_scores.append(np.sqrt(d2.min()))
maha_scores = np.array(maha_scores)


validity = np.where(maha_scores <= tau_maha,
                    "In class Distribution",
                    "Out of Distribution")


# Suppress predicted labels for OOD
final_preds = [lbl if v.startswith("In") else "N/A"
               for lbl, v in zip(pred_labels, validity)]


patient_seq["Predicted_Genotype"] = final_preds
patient_seq["Confidence"] = np.round(confidences, 6)
patient_seq["Mahalanobis"] = np.round(maha_scores, 6)
patient_seq["Validity"] = validity

patient_seq
```

Out[144]:

|  | name | sequence | Query\_Sequence\_length | Predicted\_Genotype | Confidence | Mahalanobis | Validity |
| --- | --- | --- | --- | --- | --- | --- | --- |
| 0 | patientAA | ACGCGCAGGCCGGAGGCTGGCCCCCCACGATAGGGCCAAACGTCCG... | 593 | Trichophyton indotineae | 0.999917 | 0.000001 | In class Distribution |
| 1 | patientAB | ACGCGCAGGCCGGACGCTGGCCCCCCACGATAGGGCCAAACGTCCG... | 594 | Trichophyton mentagrophytes IV | 0.999982 | 0.000004 | In class Distribution |
| 2 | patientAC | ACGCGCAGGCCGGAGGCTGGCCCCCCACGATAGGGCCAAACGTCCG... | 593 | Trichophyton indotineae | 0.999917 | 0.000001 | In class Distribution |
| 3 | patientAD | ACGCGCAGGCCGGAGGCTGGCCCCCCACGATAGGGCCAAACGTCCG... | 593 | Trichophyton indotineae | 0.999917 | 0.000001 | In class Distribution |
| 4 | patientAE | ACGCGCAGGCCGGAGGCTGGCCCCCCACGATAGGGCCAAACGTCCG... | 593 | Trichophyton indotineae | 0.999917 | 0.000001 | In class Distribution |
| 5 | patientAF | ACGCGCAGGCCGGAGGCTGGCCCCCCACGATAGGGCCAAACGTCCG... | 593 | Trichophyton indotineae | 0.999917 | 0.000001 | In class Distribution |
| 6 | patientAG | ACGCGCAGGCCGGAGGCTGGCCCCCCACGATAGGGCCAAACGTCCG... | 593 | Trichophyton indotineae | 0.999917 | 0.000001 | In class Distribution |
| 7 | patientAH | ACGCGCAGGCCGGAGGCTGGCCCCCCACGATAGGGCCAAACGTCCG... | 593 | Trichophyton indotineae | 0.999917 | 0.000001 | In class Distribution |
| 8 | patientAI | ACGCGCAGGCCGGAGGCTGGCCCCCCACGATAGGGCCAAACGTCCG... | 593 | Trichophyton indotineae | 0.999917 | 0.000001 | In class Distribution |
| 9 | patientAJ | ACGCGCAGGCCGGAGGCTGGCCCCCCACGATAGGGCCAAACGTCCG... | 593 | Trichophyton indotineae | 0.999917 | 0.000001 | In class Distribution |
| 10 | patientAK | ACGCGCAGGCCGGAGGCTGGCCCCCCACGATAGGGCCAAACGTCCG... | 593 | Trichophyton indotineae | 0.999917 | 0.000001 | In class Distribution |
| 11 | patientAL | ACGCGCAGGCCGGAGGCTGGCCCCCCACGATAGGGCCAAACGTCCG... | 593 | Trichophyton indotineae | 0.999917 | 0.000001 | In class Distribution |
| 12 | patientAM | ACGCGCAGGCCGGACGCTGGCCCCCCACGATAGGGCCAAACGTCCG... | 594 | Trichophyton mentagrophytes IV | 0.999982 | 0.000004 | In class Distribution |
| 13 | patientAN | GCGCGCAGGCCGGAGGCTGGCCCCCCACGATAGGGCCAAACGTCCG... | 593 | Trichophyton interdigitale II | 0.965935 | 0.000002 | In class Distribution |
| 14 | patientAO | ACGCGCAGGCCGGAGGCTGGCCCCCCACGATAGGGCCAAACGTCCG... | 593 | Trichophyton indotineae | 0.999917 | 0.000001 | In class Distribution |
| 15 | patientAP | ACGCGCAGGCCGGAGGCTGGCCCCCCACGATAGGGCCAAACGTCCG... | 593 | Trichophyton indotineae | 0.999917 | 0.000001 | In class Distribution |
| 16 | patientAQ | GCGCGCAGGCCGGAGGCTGGCCCCCCACGATAGGGCCAAACGTCCG... | 593 | Trichophyton interdigitale II | 0.965935 | 0.000002 | In class Distribution |
| 17 | patientAR | GCGCGCAGGCCGGAGGCTGGCCCCCCACGATAGGGCCAAACGTCCG... | 593 | Trichophyton interdigitale II | 0.965935 | 0.000002 | In class Distribution |
| 18 | patientAS | ACGCGCAGGCCGGAGGCTGGCCGCCCACGATAGGGCCAAACGTCCG... | 593 | Trichophyton mentagrophytes VII | 1.000000 | 0.000006 | In class Distribution |
| 19 | patientAT | ACGCGCAGGCCGGAGGCTGGCCCCCCACGATAGGGCCAAACGTCCG... | 593 | Trichophyton indotineae | 0.999917 | 0.000001 | In class Distribution |
| 20 | patientAU | ACGCGCAGGCCGGAGGCTGGCCCCCCACGATAGGGCCAAACGTCCG... | 593 | Trichophyton indotineae | 0.999917 | 0.000001 | In class Distribution |
| 21 | patientA | GCGCGCAGGCCGGAGGCTGGCCCCCCACGATAGGGCCAAACGTCCG... | 593 | Trichophyton interdigitale II | 0.965935 | 0.000002 | In class Distribution |
| 22 | PatientBA | TGATTCAAAACAAAAACAAGTCAAAACTTTTAACAACGGATCTCTT... | 362 | N/A | 0.619690 | 95.362183 | Out of Distribution |
| 23 | PatientBB | ACGCGCAGGCCGGAGGCTGGCCCCCCACGATAGGGACCGACGTTCC... | 620 | N/A | 0.500891 | 57.671661 | Out of Distribution |
| 24 | patientBC | TAACAAGGTTTCCGTAGGTGAACCTGCGGAAGGATCATTACCGAGT... | 548 | N/A | 0.999845 | 88.965630 | Out of Distribution |

#### Genotype assignment was based on the Mahalanobis distance (d). Values from 0 to 0.000012 were treated as perfect matches. For 0.000012 < d <= 2, the model reported the nearest (most closely related) genotype. For d > 2, samples were considered out of distribution; no genotype was assigned and the case was flagged for manual review.¶
