# Supplementary material for: The emergence of superficial dermatophytosis due to Trichophyton indotineae and Trichophyton mentagrophytes genotypes VII and II* in New York: a need for comprehensive testing approaches
Source: J Clin Microbiol. 2026 Apr 10;64(5):e00156-26. doi: 10.1128/jcm.00156-26 (PMC13170464; doi:10.1128/jcm.00156-26)
Supplement: File S2 — Details on modeling and WGS. [file jcm.00156-26-s0002.docx]

**Supplementary File 2**

**SUPPLEMENTARY MATERIALS AND METHODS**

**Automated pipeline for *Trichophyton* species and genotype determination:**

An ITS-based deep learning model for rapid and accurate *Trichophyton* species and genotype identification was developed using 28 curated reference ITS sequences from the *TiTm*SC containing six genotypes of *T. interdigitale,* 21 genotypes of *T. mentagrophytes,* and *T. indotineae* (formerly known as *T. mentagrophytes* VIII) (1). The detailed steps required for feature engineering, training and prediction of the model are provided in Supplementary Figure 2a. In brief, for feature engineering, we computed a 6-mer count of tokens for each of the 28 *TiTm*SC ITS sequences. The choice of 6-mers was based on previous findings that 6-mers are the minimum length that captures informative signals for fungal ITS classification (2). These 6-mer counts were then normalized using standard z-score scaling to ensure that no k-mer frequency dominated and to ensure stable, efficient, and balanced learning across features, making the training process faster and more reliable. For making Convolution Neural Network (CNN) model architecture, an input layer was followed by two convolutional layers with Rectifier Linear Unit (ReLU) activation, a flattening layer, a fully connected dense layer with ReLU activation, and a final output layer with SoftMax activation across 28 *TiTm*SC ITS sequences. For model hyperparameters, we used categorical cross-entropy loss to optimize 28 *TiTm*SC ITS sequences. Adam optimizer with a fixed learning rate of 3 × 10⁻⁴ and batch size of 10, and finally early stopping was applied to prevent overfitting. Once the architecture and hyperparameters were completed, the 6-mer count vector from feature engineering was used as the input feature for training the CNN classifier. Upon training, the model learned a set of weights and biases, making it ready for predictions.

Output chromatogram files (.ab1) from Sanger sequencing of the ITS region with forward (V1827) and reverse (V50) primers were directly uploaded into this pipeline. Base calls and per-base phred scores were extracted with Biopython (SeqIO), low quality bases were removed from both the 5’ and 3’ ends using a sliding window approach (window size = 5 bps) with a pred quality score cutoff off ≥ 8. Reverse strands were complemented and aligned to the forward sequence using Python library Pairwise Aligner to generate a consensus sequence. Finally, for the prediction pipeline, each input sequence was subjected to trimming of the conserved 18S and 28S sequences, by searching for conserved motifs at the 5’ ([GA]CGCGCAGGCCGGA[GC]GCTGGCC[GC]CCCACGA) and 3’ (GGCCTCA[AG]AATCTGTTTTATACTTAT[TC][GA]) edges of the ITS region (Supplementary Figure 1). The full-length ITS sequence was vectorized using the fitted 6-mer vocabulary and scaled to generate standardized feature representations. The count vectors were then fed into the CNN model to provide species and genotype identification with a confidence score (0.982 to 1.0) computed from the SoftMax probabilities. To minimize the risk of incorrect genotype assignment, sequences passed to the model that do not belong to any known reference genotype were filtered out using an out-of-distribution detection method (3). For each sequence, we computed the Mahalanobis distance to all classes and only accepted sequences below a threshold of 1. If there is a single SNP from one of the 28 genotypes, the pipeline will provide the most closely related genotype and the SNP position. Any close matches with more than one SNP will not pass the Mahalanobis threshold and give the output “Not a member of the *TiTm*SC, check sequences manually”. If the full-length ITS sequence was not obtainable, the pipeline will give the output “Insufficient sequence for genotyping”. The pipeline code can be found in Supplementary File 1.

**Confirming Species and Genotype of Reference Genomes in GenBank**

To speciate and genotype reference genomes in GenBank labeled as *Ti*, *T. mentagrophytes* or *T. interdigitale,* the consensus ITS sequence from a *TiTm*SC alignment was used as a query to extract ITS sequences from all GenBank reference genomes (when sufficient sequence was available in the ITS region of the assemblies). ITS sequences were aligned manually as well as run through the *TiTm*SC genotyping pipeline to correctly identify the species and genotype for each assembly (Supplementary Table 4).

**Determining Appropriate Reference Assemblies for Read Mapping**

A k-mer analysis of representative genomes from our collection was prepared with *Trichophyton* genomes available in GenBank using a k-mer length of 16 and a prefix of ATGAC on both strands with the Feature Frequency Profile via Jensen-Shannon divergences statistical method. In addition, our *de novo* assemblies were aligned to representative assemblies from GenBank using a minimum initial seed length of 15 (allowing for mismatches) and a minimum alignment block length of 100. Subsequently, the average nucleotide identity (ANI) was determined with a minimum similarity fraction of 0.8 and minimum length fraction of 0.8.

**Calculating SNP differences among *TiTm*SC clades**

To determine ballpark SNP differences between different *Trichophyton* genotypes, a representative from each of the seven genotypes that we have received was sequenced, mapped to the reference strain TIMM20114, and SNP trees were generated as described in the main text.

**SUPPLEMENTARY RESULTS**

**Confirming GenBank Assembly Species and Genotypes**

The *Trichophyton* genome assemblies currently available in GenBank (as of 02/01/2026 are only identified to the species level, and no genotype information is available. We downloaded all genome assemblies of *T. indotineae* (total number 19), *T. mentagrophytes* (total number 18, excluding the contaminated assembly TIMM 2789, GenBank GCA_003118255.1), and *T. interdigitale* (total number 23). The consensus *TiTm*SC ITS sequence was used to BLAST each genome assembly to extract the ITS region. ITS sequences were manually analyzed and run through our genotyping pipeline with 100% concordance. The results are summarized in Supplementary Table 4. Our genotyping revealed that all *Ti* assemblies in GenBank were correctly identified except one strain with 3 SNPs in the ITS sequence. For *T. mentagrophytes* assemblies, only 10/17 were identified as the correct species (five were*Tm*III*, two were *Tm*II* and one each was *Tm* IV, VI, and VII), 1/17 was *Ti*, 3/17 were *T. interdigitale* II and 3/17 did not have sufficient assembled sequence in the ITS region for species/genotype identification. For *T. interdigitale* assemblies, 14/23 were correctly identified as *T. interdigitale* II, 3/23 were *Ti*, 1/23 was *Tm*VII, and 5/23 did not have sufficient assembled sequence in the ITS region for species/genotype identification.

**Reference Assembly for Read Mapping**

To choose an appropriate reference strain for read mapping, we generated a k-mer tree with a representative member from each genotype of the *TiTm*SC that we have sequenced, the recommended genome assemblies from GenBank for *Ti* (TIMM20114), *T. mentagrophytes* (LL2024a) and *T. interdigitale* (IGIB-BK-CI04), as well as two strains identified as *Tm*VII (M8436 and ATCC 18748) and two strains identified as *Tm*II* (D15P152 and CBS 435.73) (Supplementary Figure 3a). In addition, the ANI between each genotype was calculated (Supplementary Figure 3b).

In the k-mer tree, our Ti isolates clustered with the Ti reference strain TIMM20114, with an ANI of 99.98%. This strain has been used previously for Ti WGS analyses (12, 13) and was chosen as a reference strain for *Ti*. For *Tm*VII, the GenBank assembly strain M8436 clustered closely with our TmVII isolates in the k-mer tree and had the highest ANI (99.98%) with our isolates; thus, M8436 was chosen as the reference for mapping of our *Tm*VII reads. We found two GenBank assemblies of genotype TmII*: D15P152 and CBS 435.73. However, in the k-mer analysis, our TmII* isolates clustered most closely with LL-2024a, a T. interdigitale II, rather than D15P152 or CBS 435.73, and had a higher ANI (99.93%) compared to 99.75% or 99.90%, respectively. Thus, our *Tm*II* reads were mapped to the reference LL-2024a.

**Single nucleotide polymorphisms (SNPs) between genotypes**

Lastly, we sought to determine the extent to which the TiTmSC genotypes differ from one another. We sequenced one isolate each of T. interdigitale I, T. interdigitale II, TmIII*, TmIV, and, together with a representative from our Ti, TmII*, and TmVII isolates, mapped reads to the Ti reference strain TMM20114 to get approximate SNPs between genotypes. Ballpark SNPs were then compared with the ANI determined from de novo-assembled whole -genome alignments (Supplementary Figure 3b). The most closely related TiTmSC genotypes were T. interdigitale I and II with ~1000 SNPs and an ANI of 99.97%. TmII* was 6000-7000 SNPs from T. interdigitale I and II with an ANI of 99.92-99.93%. Aside from this cluster, all other genotypes had 75,000-115,000 SNPs from other TiTmSC genotypes, with an average ANI of 99.27%. For context, T. rubrum, which is not a member of the TiTmSC, had ~1,400,000 SNPs with an average ANI of 90.98% from all TiTmSC genotypes analyzed.

**DISCUSSION**

Given the complexity of distinguishing among TiTmSC members, it is unsurprising that none of the genome assemblies in GenBank have been identified to the genotype level, except Ti, and many described as T. interdigitale or T. mentagrophytes have the wrong species name (Supplementary Table 4). We have used our pipeline to accurately assign species and genotypes to the T. interdigitale, T. mentagrophytes, and Ti genome assemblies in GenBank (Supplementary Table 4), making this information accessible to the broader scientific community and drawing attention to the distinct infection characteristics among TiTmSC members.

SNP differences in the ITS region alone may not be sufficient to demarcate TiTmSC species and genotypes with distinct environmental reservoirs, mechanisms of spread, host body site preferences, and antifungal drug-resistance profiles. For example, T. interdigitale infections were previously classified as exclusively anthropophilic, presenting mainly on the feet and nails. At the same time, T. mentagrophytes infections were exclusively zoophilic and affected other parts of the body (4). However, Ti (formerly T. mentagrophytes VIII) is clearly capable of anthropophilic transmission (5-7), although some cases of Ti have been identified in canines (7, 8). At the molecular level, these two species are differentiated by a single nucleotide, the presence of a “G” or an “A” at the first position of the ITS1 region (Supplementary Figure 1). It is highly unlikely that a single nucleotide in the ITS region would play a significant role in the organism’s site of infection, infection severity, host preference, or mode of transmission. Thus, further work is required to delineate which characteristics are essential for effective categorization into these two species (9).

Additionally, we performed whole-genome sequencing on one isolate that differs from TmII* in the ITS region by a single SNP (*Tm*II* G220A). The *Tm*II* G220A isolate is only 113-558 SNPs from our other *Tm*II* isolates, which is well within the range of SNPs (<687 SNPs) we see among our TmII* isolates that have a 100% match to TmII* in the ITS region. In contrast, the SNP differences among other members of the TiTmSC that we have received and sequenced are on the magnitude of 1,000-8,000 SNPs between T. interdigitale I, T. interdigitale II, and *Tm*II*; ~100,000 between other genotypes; and >1,000,000 from the outgroup T. rubrum. These SNP values are consistent with those reported by other groups in WGS analyses of members in the TiTmSC (7, 10). Therefore, ITS sequencing alone may not be sufficient to delineate new TiTmSC genotypes. Whole-genome sequencing to determine ANI and relatedness to other members of the TiTmSC, along with unique clinical presentations or resistance patterns, will be critical before it is possible to demarcate additional genotypes in the *TiTmS*C.

1. Normand AC, Moreno-Sabater A, Jabet A, Hamane S, Cremer G, Foulet F, Blaize M, Delliere S, Bonnal C, Imbert S, Brun S, Packeu A, Bretagne S, Piarroux R. 2022. MALDI-TOF mass spectrometry online identification of *Trichophyton indotineae* using the MSI-2 application. J Fungi (Basel) 8(10):1103.

2. Gunasekaran H, Ramalakshmi K, Rex Macedo Arokiaraj A, Deepa Kanmani S, Venkatesan C, Suresh Gnana Dhas C. 2021. Analysis of DNA sequence classification using CNN and hybrid models. Computational and mathematical methods in medicine 2021:1835056.

3. Anthony H, Kamnitsas K. On the use of mahalanobis distance for out-of-distribution detection with neural networks for medical imaging, p 136-146. *In* (ed), Springer,

4. Klinger M, Theiler M, Bosshard PP. 2021. Epidemiological and clinical aspects of *Trichophyton mentagrophytes/Trichophyton interdigitale* infections in the Zurich area: a retrospective study using genotyping. J Eur Acad Dermatol Venereol 35:1017-1025.

5. Caplan AS, Todd GC, Zhu Y, Sikora M, Akoh CC, Jakus J, Lipner SR, Graber KB, Acker KP, Morales AE, Rolón RMM, Westblade LF, Fonseca M, Cline A, Gold JAW, Lockhart SR, Smith DJ, Chiller T, Greendyke WG, Manjari SR, Banavali NK, Chaturvedi S. 2024. Clinical course, antifungal susceptibility, and genomic sequencing of *Trichophyton indotineae*. JAMA Dermatol 160:701-709.

6. Spivack S, Gold JAW, Lockhart SR, Anand P, Quilter LAS, Smith DJ, Bowen B, Gould JM, Eltokhy A, Gamal A, Retuerto M, McCormick TS, Ghannoum MA. 2024. Potential sexual transmission of antifungal-resistant *Trichophyton indotineae*. Emerg Infect Dis 30:807-809.

7. Thakur S, Spruijtenburg B, Abhishek, Shaw D, de Groot T, Meijer EFJ, Narang T, Dogra S, Chakrabarti A, Meis JF, Rudramurthy SM. 2025. Whole genome sequence analysis of terbinafine resistant and susceptible *Trichophyton* isolates from human and animal origin. Mycopathologia 190:13.

8. Rudramurthy SM, Shaw D, Shankarnarayan SA, Abhishek, Dogra S. 2023. Comprehensive taxonomical analysis of *Trichophyton mentagrophytes/interdigitale* complex of human and animal origin from India. J Fungi 9:577.

9. Chowdhary A, Singh A, Singh PK, Khurana A, Meis JF. 2019. Perspectives on misidentification of *Trichophyton interdigitale/Trichophyton mentagrophytes* using internal transcribed spacer region sequencing: Urgent need to update the sequence database. Mycoses 62:11-15.

10. Kumar P, Ramachandran S, Das S, Bhattacharya SN, Taneja B. 2023. Insights into changing dermatophyte spectrum in India through analysis of cumulative 161,245 cases between 1939 and 2021. Mycopathologia 188:183-202.
