# Supplementary figures and images for: The emergence of superficial dermatophytosis due to Trichophyton indotineae and Trichophyton mentagrophytes genotypes VII and II* in New York: a need for comprehensive testing approaches
Source: J Clin Microbiol. 2026 Apr 10;64(5):e00156-26. doi: 10.1128/jcm.00156-26 (PMC13170464; doi:10.1128/jcm.00156-26)

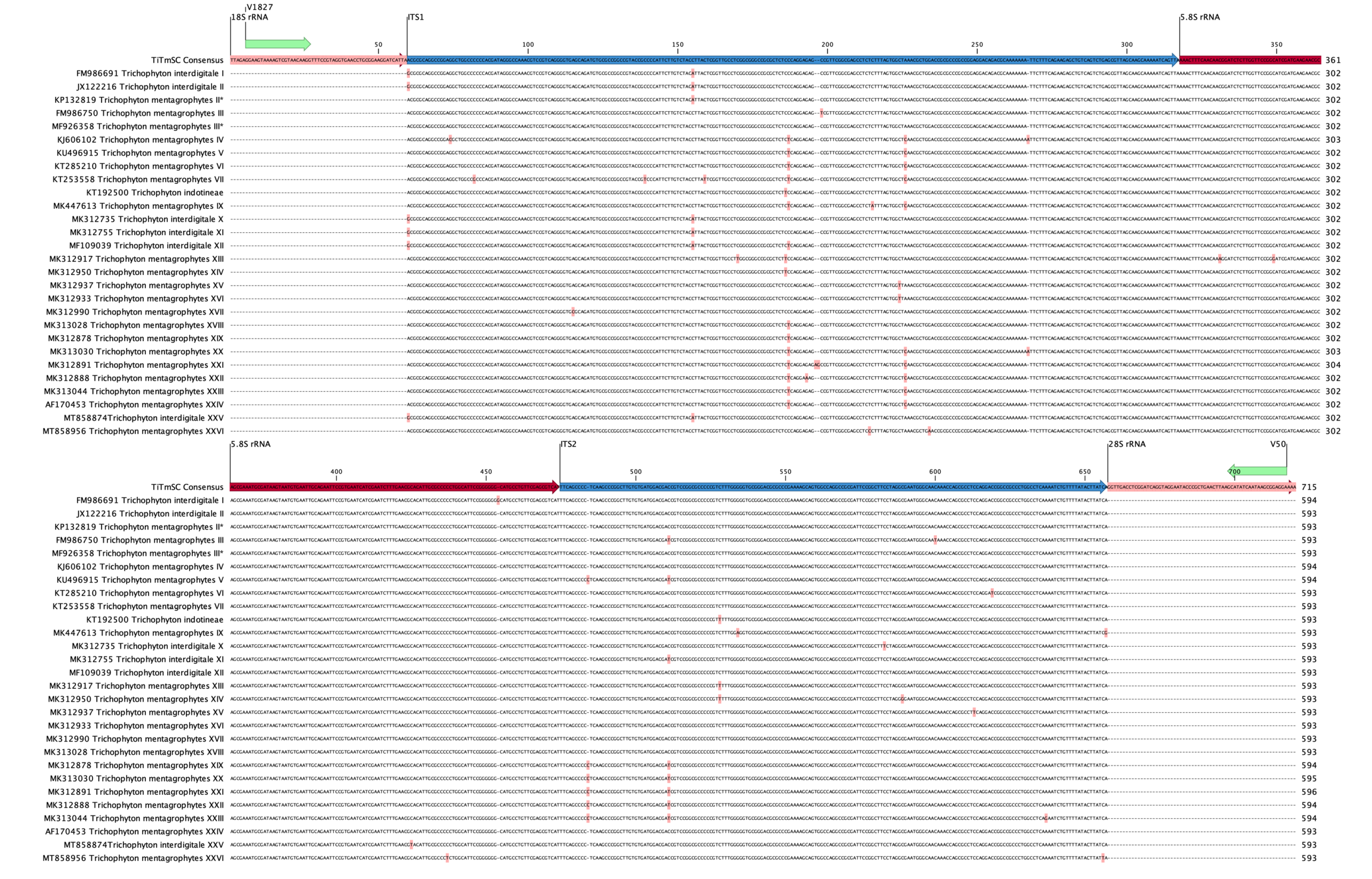

Supplement: Figure S1 — Alignment of ITS sequences for the 28 genotypes of the TiTmSC. [file jcm.00156-26-s0003.tif]

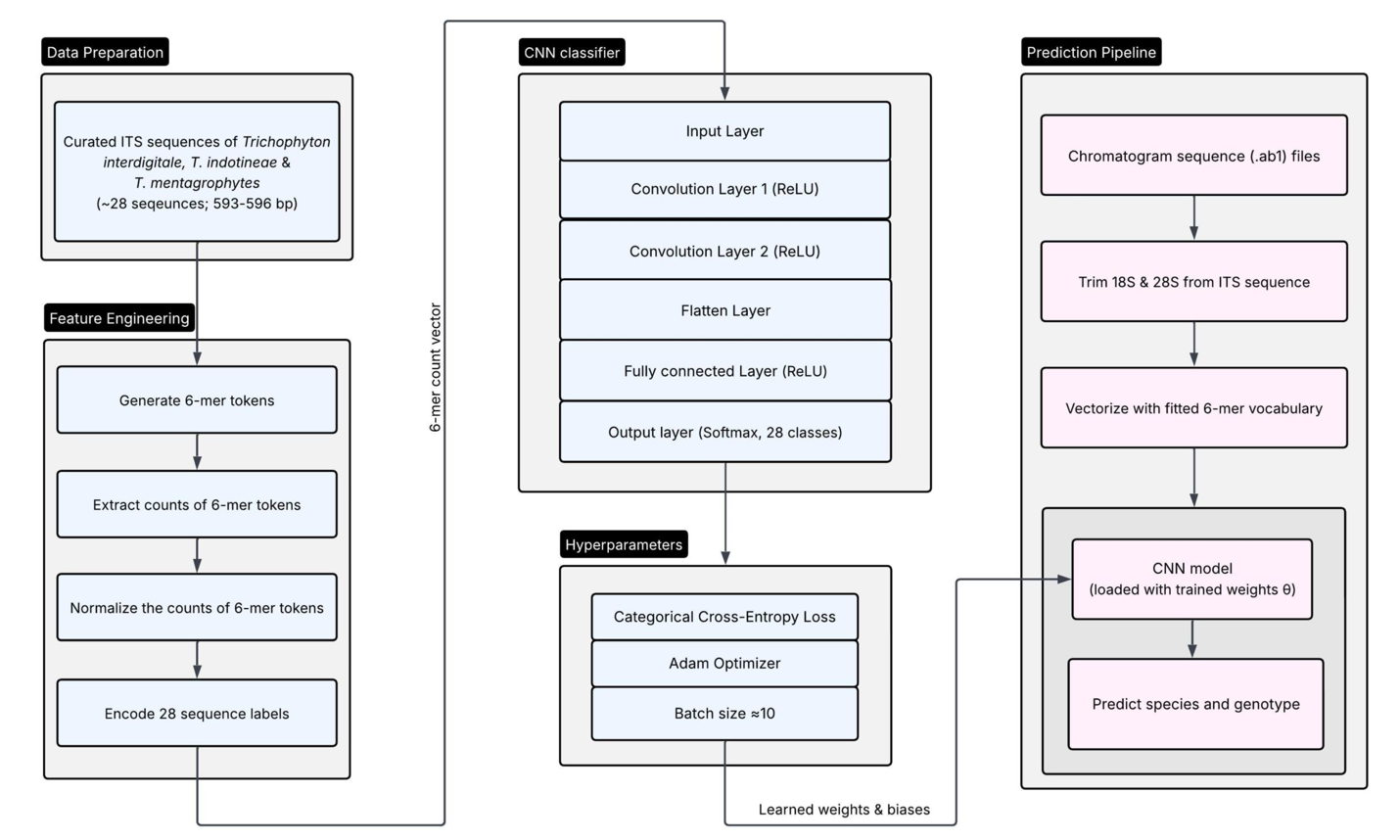
Supplementary Figure 2a

Supplementary Figure 2b


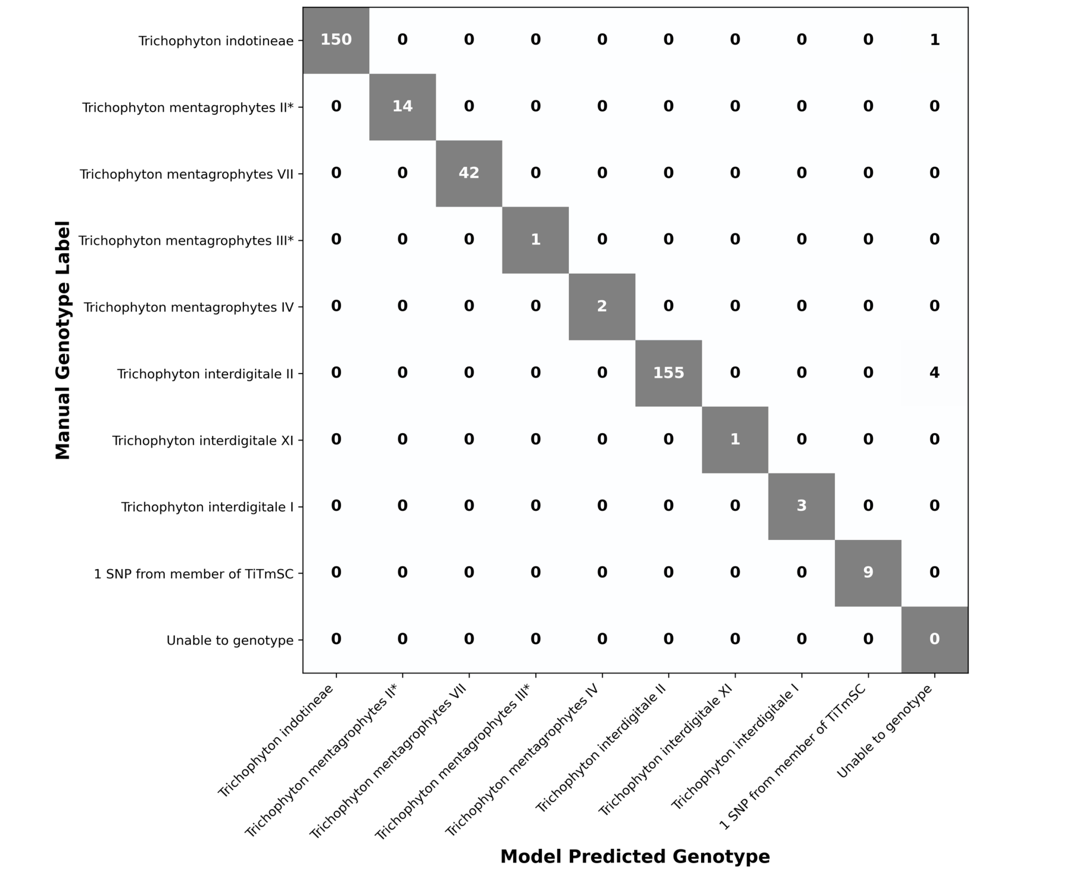


Supplementary Figure 2c


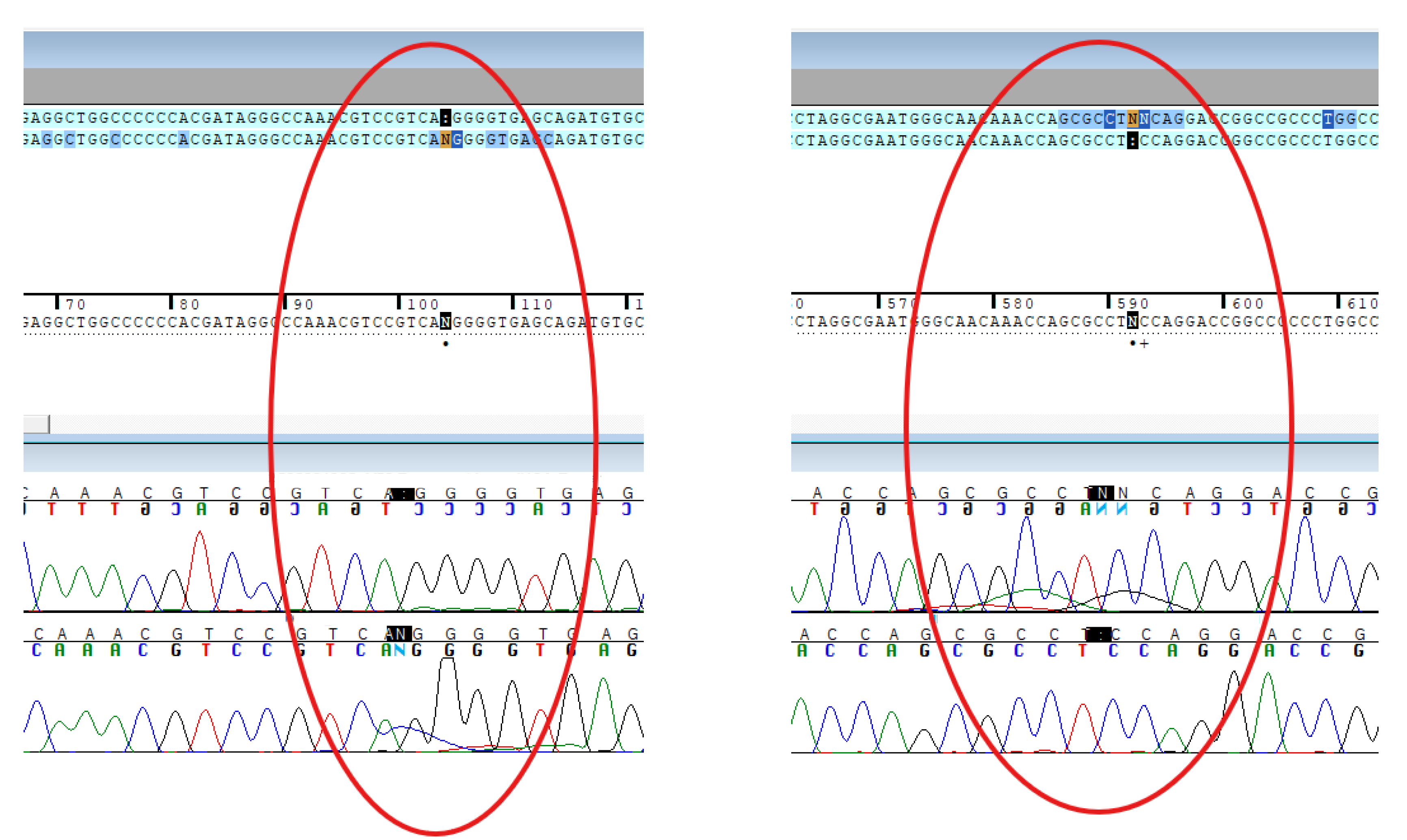

Supplement: Figures S2a-c — Pipeline analysis. [file jcm.00156-26-s0004.docx]

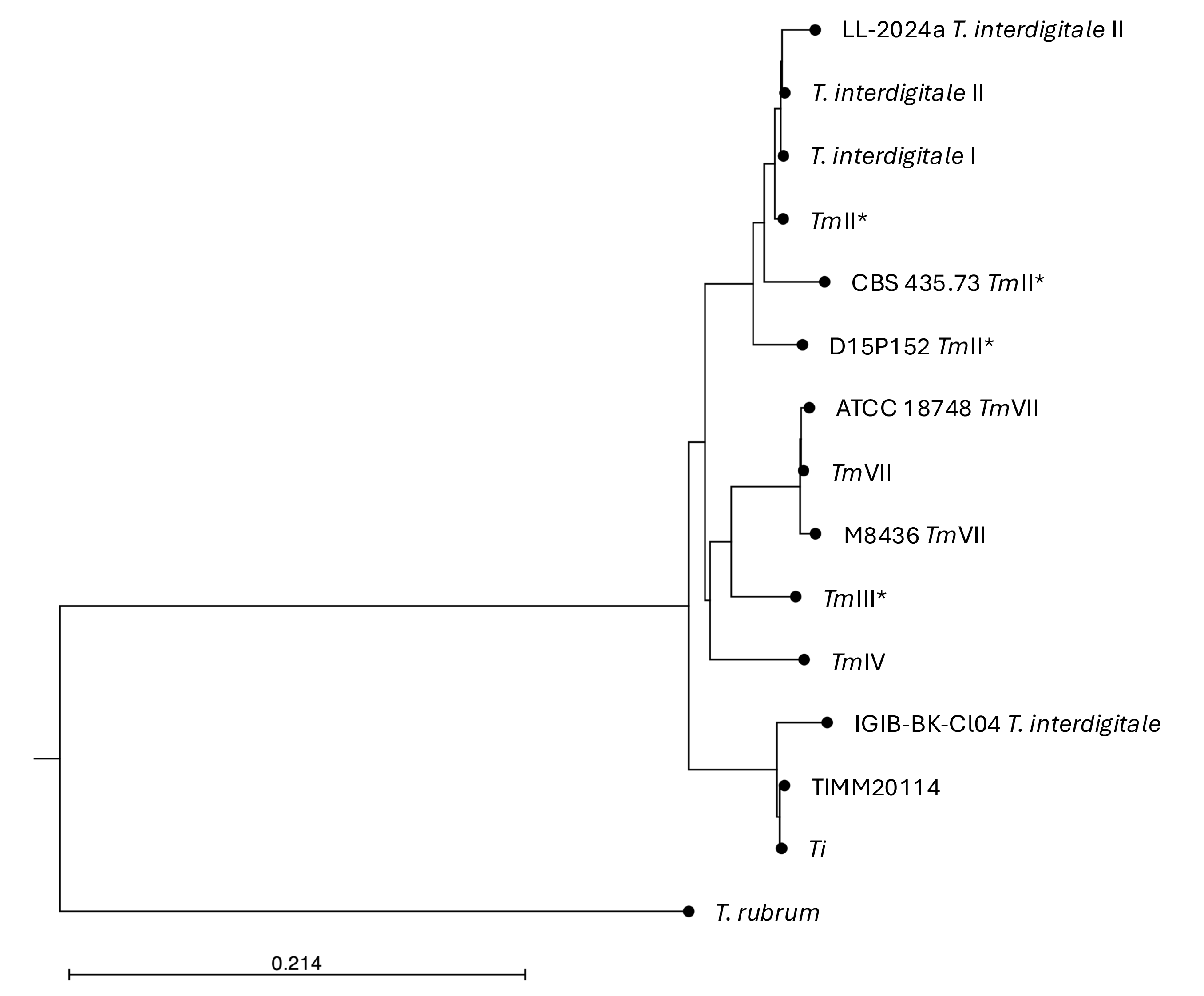

Supplement: Figure S3a — Determining the best reference strain. (a) K-mer analysis of a representative from our TiTmSC isolate assemblies along with seven GenBank assemblies. A T. rubrum isolate is included as an outgroup. [file jcm.00156-26-s0005.tif]

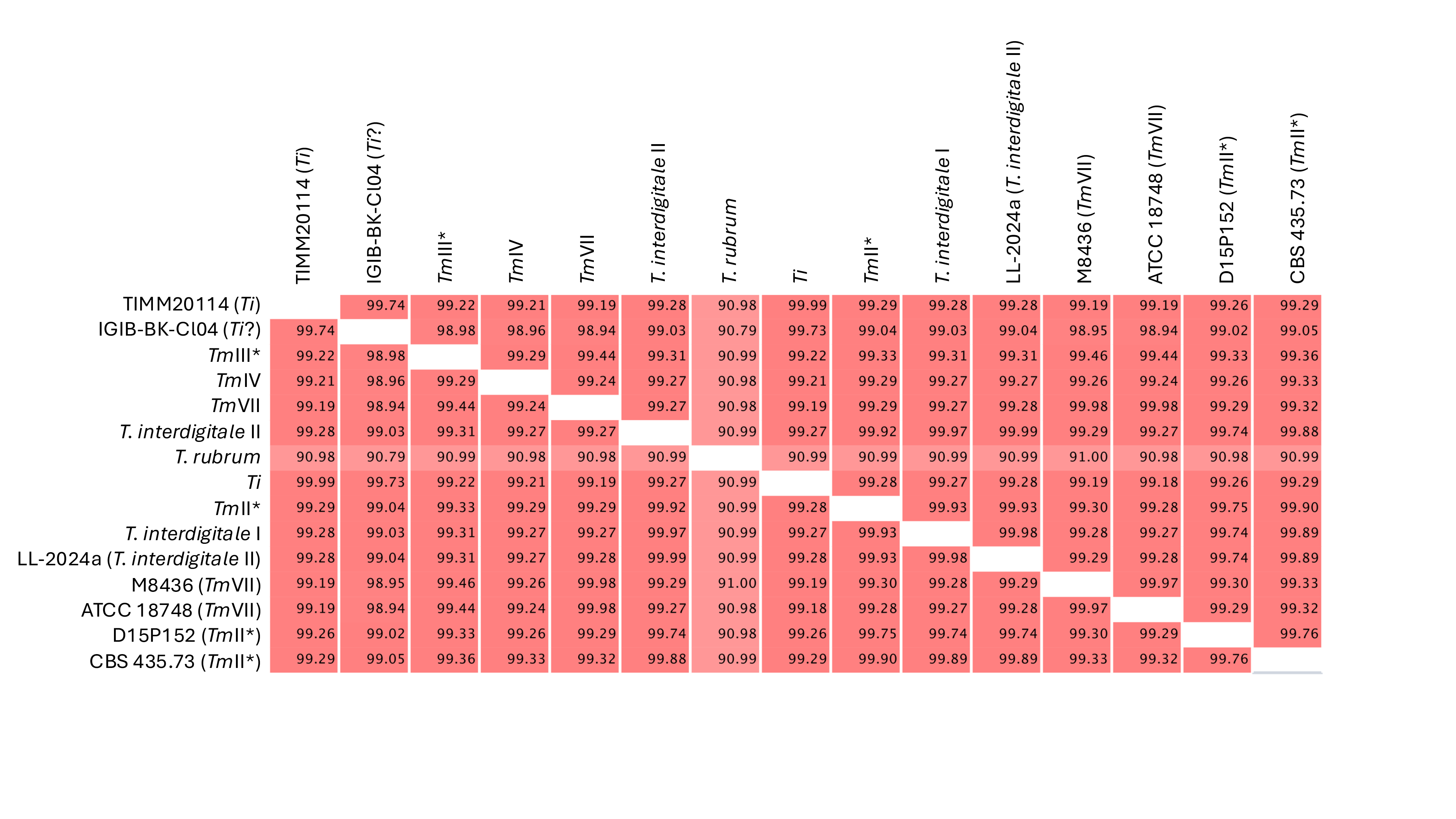

Supplement: Figure S3b — Determining the best reference strain. (b) Matrix of the average nucleotide identity (ANI) between GenBank assemblies and representatives from our TiTmSC isolate assemblies. A T. rubrum isolate is included as an outgroup. [file jcm.00156-26-s0006.tif]

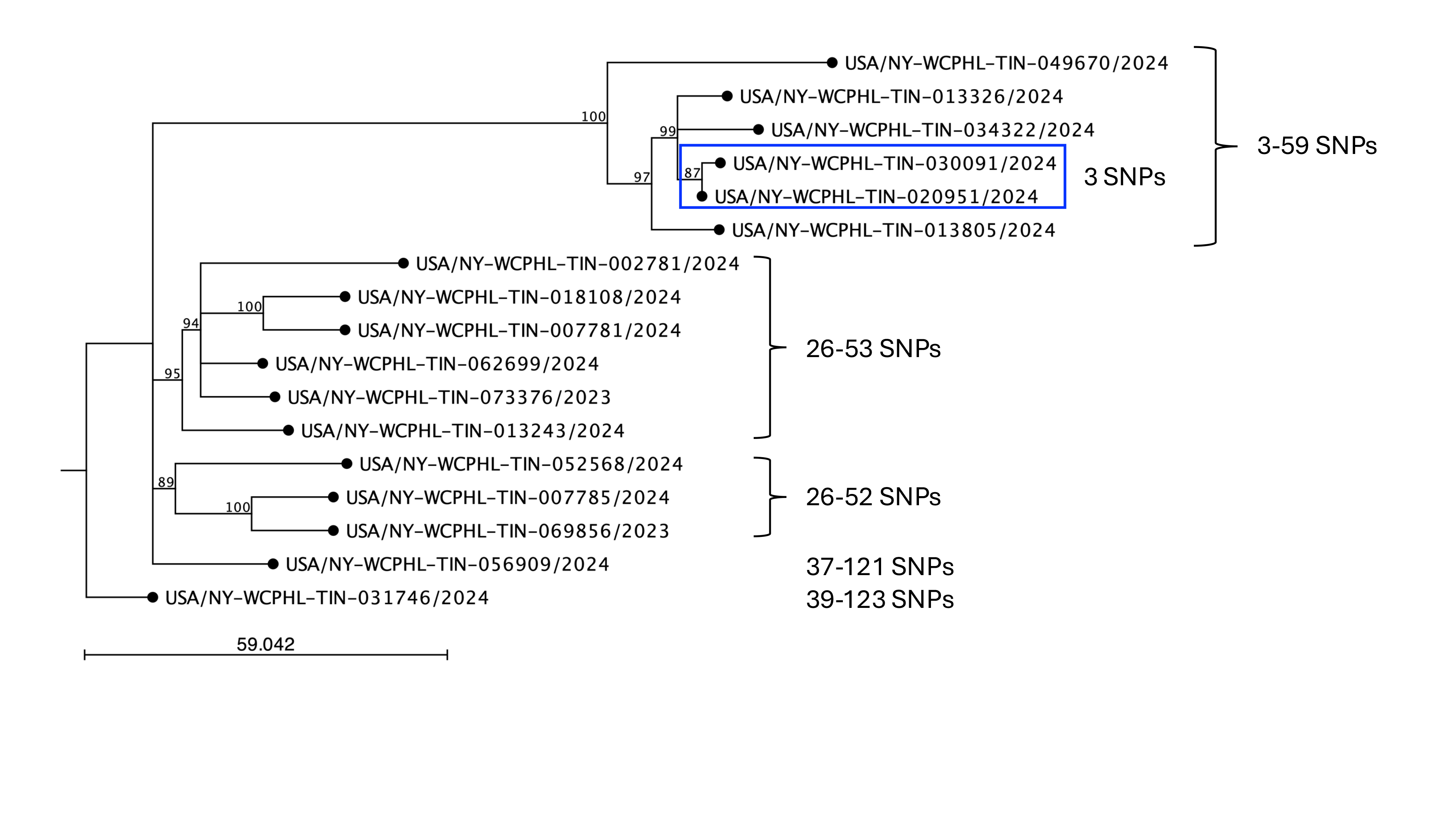

Supplement: Figure S4 — Maximum-likelihood SNP tree of Ti isolates from patients living in a single zip code. [file jcm.00156-26-s0007.tif]
