# Supplementary material for: The emergence of superficial dermatophytosis due to Trichophyton indotineae and Trichophyton mentagrophytes genotypes VII and II* in New York: a need for comprehensive testing approaches
Source: J Clin Microbiol. 2026 Apr 10;64(5):e00156-26. doi: 10.1128/jcm.00156-26 (PMC13170464; doi:10.1128/jcm.00156-26)
Supplement: Supplemental legends — Legends for Figures S1 to S4. [file jcm.00156-26-s0008.docx]

**Legends for Supplementary Figures**

**Supplementary Figure 1. Alignment of ITS sequences for the 28 genotypes of the *TiTm*SC.** The 5.8S ribosomal sequence is in red, internal transcribed spacers (ITS) 1 and 2 are in blue, and primer binding sites in conserved regions of the 18S and 28S rDNA for forward (V1827) and reverse (V50) primers, respectively, are in green. SNPs among the 28 genotypes are highlighted in pink.

**Supplementary Figure 2. Automated *TiTm*SC genotyping pipeline.** (a) Architecture of the convolutional neural network (CNN) model based ITS sequence pipeline for determination of *TiTm*SC. The diagram illustrates the end-to-end data transformation from raw chromatogram inputs to predicted genotype. (b) Confusion matrix of the convolutional neural network (CNN) model predicting *TiTm*SC. The vertical axis represents the genotype assigned through manual analysis and the horizontal axis represents the genotypes predicted by the model. There was 98.7% concordance between the two analysis methods. Diagonal entries (shaded boxes) represent isolates correctly identified by the pipeline and off-diagonal entries indicate isolates that could not be automatically genotyped via the pipeline. (c) Two examples of background in the Sanger sequencing chromatogram. In these situations, manual inspection is required for accurate genotyping.

**Supplementary Figure 3. Determining the best reference strain.** (a) K-mer analysis of a representative from our *TiTm*SC isolate assemblies along with seven GenBank assemblies. A *T. rubrum* isolate is included as an outgroup. (b) Matrix of the average nucleotide identity (ANI) between between GenBank assemblies and representatives from our *TiTm*SC isolate assemblies. A *T. rubrum* isolate is included as an outgroup.

**Supplementary Figure 4. Maximum likelihood SNP tree of *Ti* isolates from patients living in a single zip code.** Ti reads were mapped to the reference strain TIMM20114, and a phylogenetic tree was constructed using a maximum likelihood algorithm with a Jukes-Cantor substitution model and 1000 bootstrap replicates. A bootstrap cutoff of 80% was used and bootstrap values are indicated at nodes. The scale bar represents the number of expected substitutions between samples. SNP ranges in each cluster are provided next to the brackets on the right, and an instance of likely local transmission is boxed in blue and labeled with the SNP differences between those isolates.
